# Psychosocial interventions for families with minor children affected by parental cancer: An umbrella review

**DOI:** 10.1007/s00520-026-10999-y

**Published:** 2026-07-11

**Authors:** Sofia Santos, Raquel Ribeiro, Miguel Barbosa

**Affiliations:** 1 Faculdade de Psicologia, Universidade de Lisboa, Lisboa, Portugal; 2 CICPSI, Faculdade de Psicologia, Universidade de Lisboa, Lisboa, Portugal; 3 Faculdade de Medicina, Universidade de Lisboa, Lisboa, Portugal

**Keywords:** Minor children, Parental cancer, Psychosocial interventions, Family-centered care, Umbrella review

## Abstract

**Purpose:**

Research on psychosocial interventions for families with minor children affected by parental cancer has expanded over recent decades; however, the evidence remains fragmented across multiple systematic reviews. This umbrella review aims to gather and synthesize the existing systematic reviews, with a particular focus on intervention characteristics, implementation, effectiveness, methodological limitations, and implications for future research and clinical practice.

**Methods:**

A comprehensive search of CINAHL, MEDLINE, PubMed, Web of Science, Scopus, PsycINFO, PsycArticles, Epistemonikos, and the Cochrane Database of Systematic Reviews was conducted in September 2025, including gray literature. Eligible reviews were published between 1990 and 2025 in English, Portuguese, or Spanish and had full-text availability. Methodological quality was assessed using AMSTAR-2. The review was registered in PROSPERO (ID 1139397).

**Results:**

Eight systematic reviews met inclusion criteria and were synthesized narratively due to substantial clinical and methodological heterogeneity. Across these reviews, 46 distinct psychosocial interventions were identified. Overall, interventions were associated with preliminary beneficial outcomes for children, adolescents, parents, and families, particularly in improving children’s understanding of parental illness, enhancing family communication and functioning, strengthening parenting skills, and promoting psychological well-being. However, confidence in these findings was limited by the generally low methodological quality of the included reviews and the high overlap of primary studies.

**Conclusions:**

Current evidence suggests that psychosocial interventions may support families with minor children affected by parental cancer, but conclusions remain tentative due to methodological limitations. There is a clear need for the development and rigorous evaluation of developmentally and disease-stage–sensitive interventions, supported by high-quality research and institutional collaboration. Strengthening the evidence base in this area is essential to inform clinical practice, guide supportive care services, and support policy development in psycho-oncology.

**Supplementary Information:**

The online version contains supplementary material available at 10.1007/s00520-026-10999-y.

## Background

Advances in public health and medicine, together with improvements in socioeconomic conditions, have contributed to increased life expectancy. Meanwhile, the prevalence of chronic and life-limiting conditions has also increased, making them major global causes of morbidity and mortality [1, 2]. Although chronic and life-limiting conditions such as cancer are often associated with later adulthood, recent epidemiological data show rising rates of early-onset cancers and diagnoses traditionally linked to older age occurring in younger adults [3]. Their occurrence earlier in the life cycle, during the child-rearing years, may be conceptualized as a non-normative “off-time” event that deviates from the expected life-cycle trajectory [4]. Population estimates indicate that 14–24.7% of cancer patients have minor or young adult children (≤ 25 years) [5]. In this review, “families affected by parental cancer” refers to families in which a mother or father has cancer and has at least one dependent minor child.

When cancer affects parents of minor children, its psychosocial impact extends to the entire family system and can be particularly devastating [6]. Cancer has been conceptualized as a family disease [7], owing to its impact on individual and family developmental pathways and the need for families to simultaneously manage parenting responsibilities while adapting to the evolving physical, emotional, and psychosocial demands associated with diagnosis, treatment, potential progression, and, in some cases, end of life [3, 8].

Parents with cancer who have minor children experience substantial psychological distress [9] and reduced quality of life [10]. Despite this burden, most strive to maintain effective parenting and protect their children [11–13]. Communication about the illness is often anxiety-provoking, aggravated by limited professional guidance. As a protective strategy, many parents limit or delay discussions about disease severity to preserve routines [13, 14]. Concurrently, minor children of parents with cancer face distinct informational, emotional, social, and practical needs following a cancer diagnosis, frequently requiring support from family, peers, and professionals [15, 16]. When a parent faces advanced or terminal cancer, minor children consistently prefer honest, developmentally appropriate, and ongoing information; emotionally available caregivers; involvement in illness coping; and continuity of routines and normality [17, 18].

Palliative and terminal phases appear to represent periods of heightened vulnerability for both patients and family members [19–22]. During these stages, minor children of parents with cancer are at increased risk of experiencing elevated levels of anxiety, distress, depressive symptoms, and reduced self-esteem, alongside disruptions in social relationships and normative developmental processes. Feelings of disconnection from parental support, extended family, and peers may further exacerbate psychosocial and behavioral difficulties [23–25].

Empirical findings regarding the psychosocial impact of parental cancer on children remain heterogeneous, with studies reporting both adaptive and maladaptive outcomes, some of which may have long-term implications for well-being [6, 26–32]. Nevertheless, there is broad consensus that many children’s needs remain unmet in the absence of targeted psychosocial support [16], and that family-centered interventions have the potential to mitigate adverse outcomes and promote adjustment for both children and parents [20].

Lately, clinical and research interest in psychosocial interventions for families affected by parental cancer has increased, leading to the development of a diverse range of programs. Although several systematic reviews have examined aspects of these interventions (e.g., see 15,23,33–34]), the resulting evidence base remains fragmented, and conclusions are dispersed across reviews. To date, no comprehensive umbrella review has synthesized this body of work to provide an integrated overview of available psychosocial interventions, their implementation, and their effectiveness across the parental cancer trajectory.

An umbrella review is therefore timely and necessary to integrate and critically appraise existing systematic reviews, providing a comprehensive overview of psychosocial interventions for families with minor children affected by parental cancer and identifying strengths and gaps in the evidence. By examining intervention characteristics, implementation, evaluation approaches, outcomes, and methodological limitations, this synthesis can inform clinical practice, research, and evidence-based policy in psycho-oncology and cancer palliative care.

The objectives of this umbrella review are (1) to gather and synthesize evidence from systematic reviews and meta-analyses on psychosocial interventions for families with minor children affected by parental cancer; and (2) to critically evaluate the intervention characteristics, implementation, evaluation, effectiveness, methodological limitations, and future directions of this evidence base for research and clinical practice. Specifically, this review addresses the following research questions:

1. Which systematic reviews and meta-analyses have examined psychosocial interventions for families with minor children affected by parental cancer?
2. What are the key characteristics and objectives of the interventions described in these reviews?
3. What evidence exists regarding the implementation, evaluation, and effectiveness of these interventions?
4. What methodological limitations and research gaps have been identified, and what directions are recommended for future intervention development?

## Method

This review was previously registered in PROSPERO (ID 1139397). Review methodology followed the Joanna Briggs Institute (JBI) guidance for umbrella reviews [35]. The manuscript is reported according to the Preferred Reporting Items for Systematic Reviews and Meta-analyses (PRISMA) guidelines [36].

### Eligibility criteria

#### Participants

Eligible reviews included studies involving, separately or jointly: (a) minor children (< 18 years) affected by parental cancer; (b) parents with cancer who have dependent minor children, regardless of cancer type or disease stage; and (c) family caregivers or other significant adults involved in the child’s care.

Systematic reviews focused on pediatric oncology (i.e., the child as the patient), adult children of healthy parents, parenting unrelated to parental cancer, non-oncological parental illness, caregivers of adult patients without dependent children, or non-family informal caregivers were excluded.

#### Interventions/phenomenon of interest

Psychosocial interventions (e.g., counseling, family therapy, support groups, psychoeducation, school programs, expressive therapies) addressing informational, emotional, social, spiritual, or practical needs of families affected by parental cancer across the illness trajectory—including bereavement—were eligible. Interventions could target children/adolescents, parents, or the family as a whole and aimed to promote well-being and reduce secondary psychosocial difficulties associated with parental cancer. Systematic reviews exclusively examining pharmacological or medical interventions, mental health interventions unrelated to parental cancer, grief unrelated to cancer-related parental loss, marital interventions unrelated to parenting, or models of care organization were excluded.

#### Outcomes

All relevant outcomes of psychosocial interventions were considered, including but not limited to child and adolescent mental health, adjustment, quality of life, family communication and functioning, parenting skills, coping, and anticipatory or post-bereavement grief.

#### Context

Systematic reviews conducted in any cultural or healthcare context and across all stages of the parental cancer trajectory were eligible, irrespective of intervention modality or delivery setting.

#### Types of studies

Only systematic reviews—quantitative, qualitative, or mixed-methods—with or without meta-analysis were included. Systematic reviews were defined as studies using explicit, systematic methods to identify, appraise, and synthesize primary research addressing a clearly formulated research question [38]. Narrative, integrative, scoping, critical, rapid, and other types of non-systematic reviews, as well as primary studies, were excluded. Eligible gray literature was included when methodological criteria for systematic reviews were met.

### Information sources

A comprehensive search was conducted in September 2025 in the following databases: CINAHL, MEDLINE, PubMed, Web of Science, Scopus, PsycINFO and PsycArticles; Epistemonikos, and the Cochrane Database of Systematic Reviews.

Gray literature searches were performed in ProQuest Dissertations and on relevant oncology and palliative care organizations’ websites, including the European Association for Palliative Care (EAPC), the International Psycho-Oncology Society (IPOS), the Portuguese Association for Palliative Care (APCP), and the Portuguese League Against Cancer (LPCC), following the same eligibility criteria. Manual searching was also conducted.

### Search strategy

The search strategy was informed by the PICOS framework (Table 1). Outcome and comparison components were not applied in database searches to avoid unnecessary restrictions. The full search strategies are presented in Online Resource 1. Reviews published since 1990 [39, 40] in English, Portuguese, or Spanish with full-text availability were considered.

**Table 1:** Research strategy based on the PICOS approach

PICOS	Search terms
Population, patient, or problem	Child* OR preschool* OR toddlers OR infants OR kid OR dependent children OR young OR adolesce* OR teens OR secondary students OR youth OR minor children OR childhood OR youth OR son OR daughter OR tweens
	OR Parent* OR mother* OR father* OR caregiver* OR caretaker* OR famil*
	AND Neoplasm OR cancer OR oncology OR palliative care OR terminal care
	NOT
	Pediatric cancer OR adolescent cancer OR pediatric palliative care OR children hospice OR perinatal palliative care
Intervention	Support OR intervention* OR strategies OR “best practices” OR treatment OR therapy OR program OR counseling OR psychotherapy OR psychosocial OR psychoeducation OR psychology OR grie* OR berev* OR mourn* OR effectiveness OR efficacy
Comparison	Control group OR treatment as usual
Outcomes	Mental health OR communication OR support OR caregiver-child relationship OR parental competence OR emotional regulation OR adjustment OR anticipatory grief OR post-death grief
Study design	S Systematic review OR meta-analysis

*Note*: The asterisk (*) indicates truncation and was used in the electronic database searches to retrieve terms sharing the same word stem

### Screening and studies selection

Screening was performed using Rayyan software [41]. Duplicates were removed by one reviewer. Two reviewers (SS and RR) independently assessed the titles/abstracts (first level) and full texts (second level), applying eligibility criteria. Disagreements were resolved by consensus or, when necessary, a third reviewer (MG).

Overlap of primary studies across included systematic reviews was assessed using the Graphical Representation of Overlap for OVERviews (GROOVE) tool [42]. Corrected Coverage Area (CCA) values were interpreted as slight (0–5%), moderate (6–10%), high (11–15%), or very high (> 15%). All eligible reviews were retained regardless of overlap to ensure transparency and completeness.

### Data extraction

Data were independently extracted by two reviewers using the JBI umbrella reviews extraction tool [35]. Extracted data included the following study details: objectives, participant characteristics, setting/context, description of interventions/phenomena of interest, databases used, coverage dates, number and type of primary studies, country of origin, quality appraisal instruments and rating, synthesis methods, main results/conclusions, significance/direction and heterogeneity. Discrepancies were resolved by consensus.

### Methodological quality assessment

The methodological quality of included systematic reviews was assessed using AMSTAR-2 [43]. With the exception of critical domains related to meta-analysis (items 11 and 15), all items were applied in accordance with the original guidance. Since quantitative synthesis was not performed, they were replaced by item 8 (adequate description of the included studies) and item 14 (management of heterogeneity of results). Overall confidence in review findings was categorized as high, moderate, low, or critically low following AMSTAR-2 criteria. Assessments were conducted independently by two reviewers, with disagreements resolved by consensus or a third reviewer. 

### Data synthesis

Given the clinical and methodological heterogeneity of the included reviews and interventions, findings were synthesized using narrative synthesis.

## Results

### Review selection

**Fig. 1:**
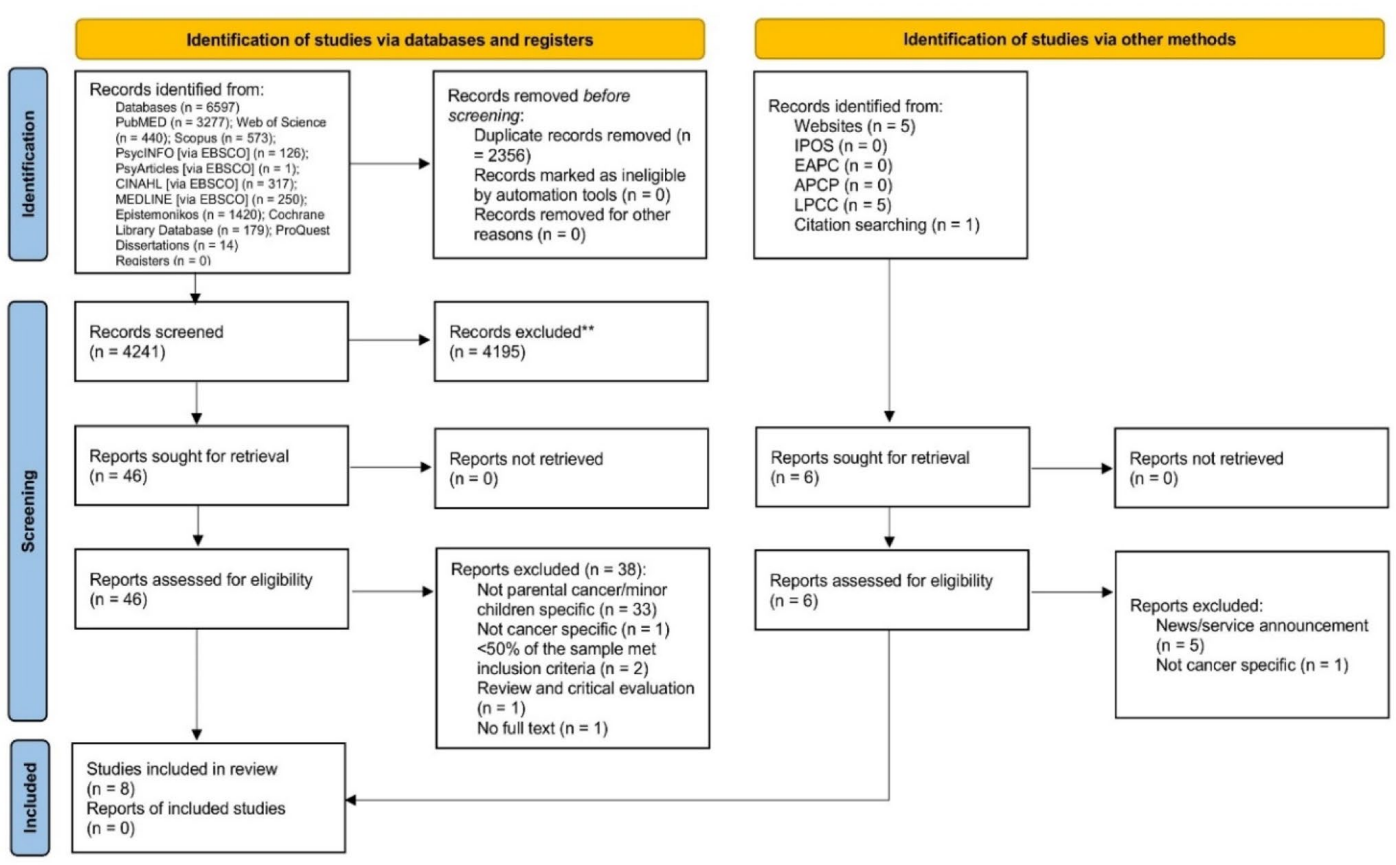
PRISMA flow diagram

Database searches yielded 4240 records after removal of 2357 duplicates (Fig. 1). Of 45 full-text articles assessed for eligibility, 37 were excluded (Online Resource 2). Searches of gray literature did not identify additional eligible systematic reviews, and one record identified through manual searching was excluded. Eight systematic reviews were included in the umbrella review.

### Overlap of primary studies

**Fig. 2:**
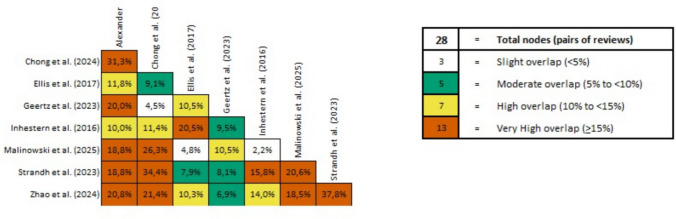
Graphical representation of overlap for Umbrella Reviews (GROOVE)

Across the eight included systematic reviews, 140 primary studies were represented, 47 of which were unique. The Corrected Covered Area (CCA) indicated a high overlap (12.03%). Pairwise overlap between reviews ranged from 2.2 to 37.8% (Fig. 2). The highest overlap (37.8%) occurred between Strandh et al. [34] and Zhao et al. [44], sharing 14 studies.

### Methodological quality of included systematic reviews

Based on the AMSTAR-2, five systematic reviews were rated low confidence and three critically low (Table 2).

**Table 2:** Methodological quality assessment of included systematic reviews using AMSTAR-2

AMSTAR-2 items	Alexander et al. [23]	Chong et al. [45]	Ellis et al. [15]	Geertz et al. [46]	Inhesternn et al. [47]	Malinowski et al. [48]	Strandh et al. [34]	Zhao et al. [44]
1. Research questions and PICO inclusion criteria components	Yes	Yes	Yes	No	Yes	Yes	Yes	Yes
2. Methods established prior to review conduct; protocol deviation justified	No	Partial Yes	No	Partial Yes	Yes	Partial Yes	Partial Yes	Partial Yes
3. Selection of study designs for inclusion explained	Yes	No	No	No	Yes	No	Yes	No
3. Comprehensive literature search strategy	Partial Yes	Partial Yes	Partial Yes	Partial Yes	Partial Yes	Partial Yes	Partial Yes	Partial Yes
5. Duplicate study selection	Yes	No	Yes	No	Yes	Yes	Yes	Yes
6. Duplicate data extraction	No	Yes	No	Yes	Yes	Yes	Yes	Yes
7. List of excluded studies and justification	No	No	No	No	No	No	No	No
8. Adequate detailed description of included studies	Yes	Partial Yes	Yes	Partial Yes	Partial Yes	Yes	Yes	Yes
9. Assessment of RoB in included individual studies	No	Partial Yes	Yes	Yes	Yes	Yes	Partial yes	Yes
10. Funding sources of the included studies	No	No	Yes	Yes	Yes	Yes	No	Yes
11. If meta-analysis: appropriate methods for statistical results combination	No meta-analysis conducted	No meta-analysis conducted	No meta-analysis conducted	No meta-analysis conducted	No meta-analysis conducted	No meta-analysis conducted	No meta-analysis conducted	No meta-analysis conducted
12. If meta-analysis: assess the potential RoB/impact of studies	No meta-analysis conducted	No meta-analysis conducted	No meta-analysis conducted	No meta-analysis conducted	No meta-analysis conducted	No meta-analysis conducted	No meta-analysis conducted	No meta-analysis conducted
13. Account of RoB of studies when interpreting/discussing the results	Yes	Yes	Yes	Yes	No	Yes	Yes	Yes
14. Satisfactory explanation for/discussion of results heterogeneity	Yes	Yes	Yes	Yes	Yes	Yes	Yes	Yes
15. If quantitative synthesis: publication bias assessed and impact discussed on the results	No meta-analysis conducted	No meta-analysis conducted	No meta-analysis conducted	No meta-analysis conducted	No meta-analysis conducted	No meta-analysis conducted	No meta-analysis conducted	No meta-analysis conducted
16. Potential conflict of interest source reported	Yes	Yes	No	Yes	Yes	Yes	Yes	Yes
Overall confidence in the review results	Critically low	Low	Critically Low	Low	Critically Low	Low	Low	Low

The most consistent methodological limitation was the absence of a list of excluded studies with justification (AMSTAR-2 item 7). Additional recurrent limitations included incomplete reporting of protocol registration or deviations, lack of duplicate study selection and/or data extraction, and variable assessment and consideration of risk of bias.

### Characteristics of included systematic reviews

The eight systematic reviews [15, 23, 34, 44–48] were published between 2016 and 2025, mainly in Germany, Australia, and China (two each), with one each from the United States and Sweden (Online Resource 3). Collectively, they included 140 primary studies (1990–2023) from North America, Europe, Asia, and Oceania. The US contributed the most (*n* = 64).

Five reviews used mixed-methods [15, 23, 34, 46, 47] and three were quantitative [44, 45, 48]. None of the included reviews conducted meta-analysis; all relied on narrative synthesis, mainly due to substantial clinical and methodological heterogeneity. Across reviews, at least 3137 participants were represented: school-aged children/adolescents (*n* = 2285), parents with cancer (*n* = 496) and other caregivers/family members (*n* = 356). Several reviews did not report total sample sizes.

Breast cancer was the most frequent diagnosis. Many studies included mixed or unspecified cancer types. Time since parental cancer diagnosis ranged from newly diagnosed to five years post-diagnosis. Most studies focused on stages 0–III. Samples generally showed limited ethnic diversity.

### Characteristics of psychosocial interventions

Given the high overlap of primary studies, a matrix was constructed to identify unique interventions. A total of 49 interventions were identified across the included reviews. Three interventions targeting healthcare professionals were excluded, leaving 46 family-focused psychosocial interventions for analysis (Online Resource 4). The main findings are summarized in Table 3.

**Table 3:** Summary of main findings by study aim

Aim	Main findings
Characteristics and objectives	**Focus and parental cancer stage:** Mainly family-focused interventions targeting school-aged children; predominantly at non-terminal cancer stages **Theoretical basis:** Primarily based on cognitive-behavioral, family systems, social-cognitive, attachment-informed, and coping-oriented approaches **Key objectives:** Emotional well-being; parenting and communication skills; illness understanding; adjustment, quality of life, and grief support
Implementation	**Components:** Psychoeducation, communication support, emotional expression, coping skills, and family functioning support **Delivery:** Heterogeneous formats, intensity, and settings (individual, group, face-to-face, online, and hybrid interventions) **Facilitators:** Delivered by trained multidisciplinary professionals
Evaluation approaches and reported effectiveness	**Evaluation approaches:** Diverse study designs and outcome measures, limiting comparability across interventions **Reported effectiveness:** Preliminary positive effects on psychological outcomes, family functioning and communication, parenting skills, disease understanding, well-being, quality of life, parental distress, and grief **Evidence strength:** Overall evidence remains limited
Methodological limitations	**Review quality:** Predominantly low or critically low confidence reviews **Heterogeneity:** Substantial variation across populations, interventions, outcomes, and study designs; no meta-analyses **Generalizability:** High study overlap, incomplete reporting, and limited cultural diversity restricted interpretation and transferability
Future directions	**Research priorities:** Stronger theoretical grounding, rigorous designs, standardized outcomes, diverse samples, and greater attention to advanced, end-of-life, and bereavement phases **Clinical and implementation priorities:** Early family-centered support, developmentally tailored interventions, and enhanced integration into routine care through multidisciplinary collaboration

#### Focus and stage of parental cancer of the interventions

Most interventions targeted the family as a whole, mainly school-aged children [44, 45, 47], followed by child-focused [15, 23, 48] and adolescent-focused [46] interventions, with fewer primarily targeting parents [34]. family-focused interventions were underrepresented during advanced disease, end-of-life, and bereavement.

#### Theoretical basis and intervention objectives

Five reviews [15, 23, 34, 45, 47] indicated that most interventions described an explicit theoretical basis, though frameworks varied, including cognitive-behavioral, family systems, social-cognitive, attachment-informed, expressive, and transtheoretical coping approaches. Others drew on clinical practice, qualitative research with families, or adaptations of models from parental mental illness.

Four reviews [15, 23, 45, 47] indicated that interventions generally aimed to improve emotional functioning, strengthen parenting skills, enhance parent–child communication, support children’s understanding of parental illness, provide opportunities for emotional expression and peer support, promote normalization and adjustment, enhance quality of life, and support anticipatory or post-bereavement grief.

### Implementation of interventions

Reviews reported substantial heterogeneity in intervention components, delivery formats, timing, and contexts, though core components were consistent.

Five reviews [15, 23, 34, 44, 48] described diverse content, including developmentally tailored psychoeducation, support for parent–child communication, emotional expression and normalization, coping skills, and strategies to sustain family functioning.

Six reviews [15, 23, 44–47] reported variation in frequency, duration, and intensity, ranging from single sessions to programs up to 22 sessions, typically lasting 6–12 weeks. Individual sessions lasted 30–120 min, except a Malta summer camp (three days/week, for eight weeks, six hours/day).

Six reviews [15, 23, 34, 44, 46, 47] described delivery modes and contexts: individual, group, or combined formats tailored to participants, with school-aged children's groups of 3–6 and adolescent/young adult groups of 8–10. Some interventions had manuals and were delivered in hospitals, outpatient clinics, homes, schools, community venues, camps, or via online, telephone, or hybrid modalities.

Five reviews [23, 34, 44, 45, 48] mention trained facilitators (e.g., therapists, nurses, psychologists, social workers, deacons). Some interventions included session evaluation (e.g., self-assessment, interviews, or video review).

#### Barriers and facilitators to implementation

One review [47] synthesized barriers and facilitators to implementation. Barriers included practical constraints (e.g., time demands, travel distance, insurance-related issues), emotional factors (e.g., perceived lack of need, burden, or stigma), disease-related characteristics (e.g., symptom burden, disease stage), and limited institutional collaboration. Facilitators included proactive information provision (e.g., referral by healthcare professionals), accessible and developmentally sensitive interventions, alignment with perceived family needs, and effective team/institutional collaboration (e.g., integration into care routine). Other reviews [34, 46] reported similar implementation challenges and facilitators, particularly in group-based interventions.

### Evaluation approaches and reported effectiveness

Across reviews, psychosocial interventions were evaluated using heterogeneous study designs, including randomized and quasi-experimental trials, feasibility and pilot studies, qualitative evaluations, and mixed-methods approaches [15, 23, 34, 44–48]. Outcomes were measured using diverse questionnaires, interviews, and combined methods, limiting comparability.

Despite these limitations, all reviews reported preliminary benefits for families affected by parental cancer. Reported outcomes included improvement in post-traumatic stress, emotional regulation, depression [15, 23, 47], anxiety, and externalizing and internalizing problems in children [15, 44, 45, 47, 48]; increased children’s knowledge of the disease, improved family functioning and communication, and enhanced parenting skills [15, 44, 47]; improved children and adolescent’s well-being and quality of life (e.g., reduced isolation, emotional expression, normalization, coping skills, and leisure opportunities) [44, 46, 47]; and improvement in parental distress [34] and grief [15, 48]. Nevertheless, overall evidence remains limited [15, 23, 34, 44–48].

## Discussion

This umbrella review critically synthesized evidence on psychosocial interventions for families with minor children affected by parental cancer across the disease trajectory, including bereavement. Drawing on eight systematic reviews, it outlines intervention characteristics, implementation, evaluation, effectiveness, methodological limitations, and gaps in the evidence.

### Interpretation of main findings

The identification of only eight eligible systematic reviews reflects both growing interest in the topic and the limited consolidated evidence available. Most were published in the past two years and originated from diverse geographical contexts, indicating increased recognition of the psychosocial impact of parental cancer when dependent children are present. This finding also highlights the urgent need for evidence-based guidelines in a context marked by clinical complexity and emotional vulnerability.

Across reviews, a broad range of psychosocial interventions was identified, with 46 family-directed interventions targeting children [15, 23, 48], adolescents [46], parents, or the family as a whole [44, 45, 47]. These interventions varied widely in theoretical orientation, objectives, delivery formats, timing, and settings, reflecting attempts to address heterogeneous needs across developmental stages and phases of the disease [34, 47]. Despite the diversity of psychosocial interventions identified, it is important to strengthen the content of parent-centered interventions [45] even in interventions predominantly aimed at children and families, not only because sick parents report high levels of distress [13], but also because healthy parents, who often take on the role of primary caregivers, express difficulty in balancing the needs of all family members [49] and request professional guidance for managing this situation [50].

Overall, the included reviews reported preliminary beneficial effects of psychosocial interventions on outcomes relevant to children, parents, and families. These included improvements in children’s emotional functioning and illness-related understanding, reductions in parental distress, and enhanced family communication [15, 44, 47]. However, these findings must be interpreted cautiously given the methodological weaknesses of the underlying evidence and the low confidence ratings assigned to most reviews.

### Focus of interventions and gaps across the disease trajectory

Most psychosocial interventions identified targeted children, adolescents, or the family as a whole, whereas comparatively few focused primarily on parents. This imbalance is noteworthy, given that both parents with cancer and healthy caregiving parents frequently report high levels of psychological distress and challenges in maintaining effective parenting under illness-related strain. Evidence suggests that parental well-being and child adjustment are closely interconnected, indicating that parent-focused support may indirectly benefit children [37]. Nevertheless, the field of psychosocial support for parents with cancer remains relatively recent, largely concentrated in Western contexts, and predominantly focused on mothers [45].

A further critical gap concerns the limited availability of interventions addressing advanced, palliative, and terminal stages of parental cancer [46, 47], as well as anticipatory and post-bereavement phases [48]. Most interventions focused on the early stages of the disease and its impact on minor children, leaving families relatively unsupported during periods of heightened vulnerability. This gap is concerning given evidence that families—and children in particular—experience substantial distress during end-of-life phases. Little is known about how families with minor children prepare for parental loss [51] and adapt to life after death [52]. Therefore, the best way to support them is unknown. Palliative care professionals have reported significant barriers to communicating with children about advanced illness, including limited training, time constraints, and lack of resources, often resulting in children being sidelined during critical periods [53–55]. They usually intervene mainly with the primary caregivers [33], relegating children to an invisible position at a time of high vulnerability [53].

### Conceptual and implementation considerations

The interventions reviewed were informed by diverse theoretical frameworks, including cognitive-behavioral, family systems, attachment-informed, and expressive approaches, as well as by clinical experience and qualitative research with affected families [34, 47]. However, several reviews highlighted the absence of clearly articulated developmental or implementation frameworks guiding intervention design [23]. This lack of conceptual integration may contribute to variability in intervention content, delivery, and evaluation, and may limit the scalability and sustainability of programs.

Interventions were implemented in multiple sessions, in a variety of settings, and by different trained professionals. Implementation challenges were commonly reported across reviews [34, 46–48] and included practical barriers for families (e.g., time demands, travel distance, insurance-related constraints), emotional barriers (e.g., stigma, perceived lack of need, fear of burden), disease-related factors, and limited institutional collaboration. Conversely, facilitating factors included proactive referral by healthcare professionals, integration of interventions into routine care, accessibility and developmental sensitivity of programs, and effective collaboration among multidisciplinary teams [47].

### Implications for intervention design and delivery

Several reviews proposed considerations for future intervention development. These included adopting flexible formats with varying duration and intensity, tailoring delivery modalities to family needs and circumstances, and combining individual and group-based approaches to accommodate heterogeneous preferences [15, 34, 45]. Interventions involving parents—even when primarily child-focused—were highlighted as potentially beneficial for enhancing parenting skills and age-appropriate communication [15, 44, 45].

Some reviews suggested that structured, evidence-informed approaches (e.g., cognitive-behavioral strategies, mindfulness-based techniques, bibliotherapy) may offer advantages over non-directive support alone [15], while still recognizing the preventive and supportive value of group-based interventions [46].

Integrative models, such as the three-component framework proposed by Malinowski et al. [48], which emphasizes education, guidance, and support/connection, offer promising conceptual directions. Such models highlight the potential value of multicomponent, developmentally sensitive interventions that can be adapted across different stages of the parental cancer trajectory and embedded within collaborative institutional frameworks.

These observations should be interpreted as emerging hypotheses rather than definitive conclusions, given the limitations of the existing evidence.

### Methodological limitations of the evidence base

The confidence in the findings of this umbrella review is constrained by several methodological limitations. Most included systematic reviews were rated as having low or critically low confidence. The high overlap of primary studies across reviews may have inflated perceptions of evidence volume and increased redundancy. However, this aspect was mitigated by the absence of meta-analyses and the explicit description of the degree of overlap.

Substantial clinical and methodological heterogeneity across primary studies—differences in populations, disease stages, intervention types, theoretical frameworks, outcome measures, and study designs—precluded quantitative synthesis and limited comparability. Incomplete reporting of participant characteristics and sociodemographic data further constrained interpretation. Additionally, the predominance of studies conducted in Western countries and limited ethnic diversity restrict the generalizability of findings to other cultural and healthcare contexts.

### Strengths and limitations of the umbrella review

This umbrella review is strengthened by its comprehensive scope, inclusion of diverse systematic reviews across a wide temporal and geographical range, and explicit assessment of overlap and methodological quality. The detailed mapping of intervention characteristics, implementation, and reported outcomes provides a nuanced overview of the current landscape of psychosocial interventions for families affected by parental cancer.

Nevertheless, several limitations should be acknowledged. Language restrictions may have resulted in the omission of relevant reviews, and unpublished interventions implemented in clinical settings may not have been captured. Duplicate removal was performed by a single reviewer, which may introduce selection bias, although this risk was mitigated through subsequent independent screening and consensus procedures.

### Recommendations for future research and clinical practice

This umbrella review identifies priorities for research and the refinement of psychosocial care of families with minor children affected by parental cancer. Intervention development, evaluation, and implementation should be strengthened, incorporating the perspectives of different family members in intervention design and theoretical model development. A deeper understanding of families’ needs and preferences across developmental stages and disease phases is essential to ensure timely, acceptable, and effective support [15, 23, 44–46].

Future research should increase methodological rigor through randomized controlled trials, standardized and developmentally sensitive outcome measures, more diverse samples, and greater inclusion of the palliative, end-of-life, and bereavement phases. Greater investment in implementation research is also needed to enhance feasibility, sustainability, and integration of psychosocial interventions into real-world care settings [15, 23, 44, 45, 48].

Clinically, families should be included early in care planning, with psychosocial interventions integrated into routine practice.

Family-centered approaches that address the needs of children, adolescents, and parents are particularly relevant. Multicomponent interventions targeting communication, psychoeducation, parenting, and coping should be tailored to developmental stage, disease phase, and sociocultural context, while accounting for practical constraints (e.g., time, accessibility, cost).

Effective delivery requires multidisciplinary collaboration and facilitators trained in psycho-oncology and family intervention. Strengthened interinstitutional collaboration may improve continuity of care, extending support beyond diagnosis and treatment to advanced disease, end-of-life, and bereavement.

## Conclusion

This umbrella review provides a comprehensive overview and critically appraises the current state of evidence on psychosocial interventions for families with minor children affected by parental cancer. Across existing systematic reviews, these interventions were associated with preliminary benefits, including children’s understanding of parental illness, family communication and functioning, parenting skills, and psychological well-being.

However, confidence in these findings is limited by the low methodological quality of the included reviews, substantial clinical and methodological heterogeneity among primary studies, and considerable overlap across reviews. These factors preclude firm conclusions about effectiveness and warrant cautious interpretation.

Despite these limitations, the findings highlight the importance of advancing and consolidating psychosocial support for this population. Greater methodological rigor, stronger theoretical grounding, and improved implementation in future research are essential to develop robust, evidence-based interventions capable of informing clinical practice, supportive care services, and health policy in psycho-oncology.

## Supplementary Information

Below is the link to the electronic supplementary material.

ESM 1 (DOCX 16.6 KB)

ESM 2 (DOCX 24.3 KB)

ESM 3 (DOCX 33.2 KB)

ESM 4 (DOCX 44.4 KB)

## Data Availability

This study is an umbrella review based exclusively on previously published systematic reviews. No new primary data were generated. All data analyzed in this study are available in the cited articles.
