# Supplementary material for: Psychosocial interventions for families with minor children affected by parental cancer: An umbrella review
Source: Support Care Cancer. 2026 Jul 11;34(8):756. doi: 10.1007/s00520-026-10999-y (PMC13356062; doi:10.1007/s00520-026-10999-y)
Supplement: Supplementary file 1 — (DOCX 16.6 KB) [file 520_2026_10999_MOESM1_ESM.docx]

**Article title:**
Psychosocial interventions focused on families with minor children affected by parental cancer: An umbrella review

**Journal:**
Supportive Care in Cancer

**Authors:**
Sofia Santos, Raquel Ribeiro, Miguel Barbosa

**Corresponding author:**
Sofia Santos

Faculty of Psychology, University of Lisbon. Lisbon. Portugal

anassantos2@edu.ulisboa.pt

**Online Resource 1**

Search strategies based on the electronic databases searched

| Electronic database | Search strategy | Applied filters |
| --- | --- | --- |
| PubMED | ((((((child*[Title/Abstract] OR preschool*[Title/Abstract] OR toddler*[Title/Abstract] OR infant*[Title/Abstract] OR kid[Title/Abstract] OR "dependent children"[Title/Abstract] OR young[Title/Abstract] OR adolesce*[Title/Abstract] OR teen*[Title/Abstract] OR "secondary students"[Title/Abstract] OR youth[Title/Abstract] OR "minor children"[Title/Abstract] OR childhood[Title/Abstract] OR son[Title/Abstract] OR daughter[Title/Abstract] OR tween*[Title/Abstract])) OR ((parent*[Title/Abstract] OR mother*[Title/Abstract] OR father*[Title/Abstract] OR caregiver*[Title/Abstract] OR caretaker*[Title/Abstract] OR famil*[Title/Abstract]))) AND ((neoplasm*[Title/Abstract] OR cancer[Title/Abstract] OR oncology[Title/Abstract] OR "palliative care"[Title/Abstract] OR "terminal care"[Title/Abstract]))) AND ((support[Title/Abstract] OR intervention*[Title/Abstract] OR strateg*[Title/Abstract] OR "best practices"[Title/Abstract] OR treatment[Title/Abstract] OR therapy[Title/Abstract] OR program*[Title/Abstract] OR counseling[Title/Abstract] OR psychotherapy[Title/Abstract] OR psychosocial[Title/Abstract] OR psychoeducation[Title/Abstract] OR psychology[Title/Abstract] OR grie*[Title/Abstract] OR bereav*[Title/Abstract] OR mourn*[Title/Abstract] OR effectiveness[Title/Abstract] OR efficacy[Title/Abstract]))) AND (("systematic review"[Publication Type] OR "systematic review"[Title/Abstract] OR "meta-analysis"[Title/Abstract]))) NOT ("pediatric cancer" OR "adolescent cancer" OR ("pediatric palliative care" OR "children hospice" OR "perinatal palliative care")) | Systematic review/1990-2025; English, Portuguese, Spanish  Search date: 02/09/2025 |
| CINAHL (EBSCOhost) | XB ((child* OR preschool* OR toddler* OR infant* OR kid OR "dependent children" OR young OR adolesce* OR teen* OR "secondary students" OR youth OR "minor children" OR childhood OR son OR daughter OR tween*)) AND XB ((parent* OR mother* OR father* OR caregiver* OR caretaker* OR famil*)) AND XB ((neoplasm OR cancer OR oncology OR "palliative care" OR "terminal care")) AND XB ((support OR intervention* OR strateg* OR "best practices" OR treatment OR therapy OR program OR counseling OR psychotherapy OR psychosocial OR psychoeducation OR psychology OR grie* OR bereav* OR mourn* OR effectiveness OR efficacy))) AND PT (("systematic review" OR "meta-analysis")) NOT TX (("pediatric cancer" OR "childhood cancer" OR "adolescent cancer" OR "pediatric palliative care" OR "children hospice" OR "perinatal palliative care")) | 1990/01/01 – 2025/09/01; English, Portuguese, Spanish  Search date: 01/09/2025 |
| MEDLINE (EBSCOhost) | XB ((child* OR preschool* OR toddler* OR infant* OR kid OR "dependent children" OR young OR adolesce* OR teen* OR "secondary students" OR youth OR "minor children" OR childhood OR son OR daughter OR tween*)) AND XB ((parent* OR mother* OR father* OR caregiver* OR caretaker* OR famil*)) AND XB ((neoplasm OR cancer OR oncology OR "palliative care" OR "terminal care")) AND XB ((support OR intervention* OR strateg* OR "best practices" OR treatment OR therapy OR program OR counseling OR psychotherapy OR psychosocial OR psychoeducation OR psychology OR grie* OR bereav* OR mourn* OR effectiveness OR efficacy))) AND PT (("systematic review" OR "meta-analysis")) NOT TX (("pediatric cancer" OR "childhood cancer" OR "adolescent cancer" OR "pediatric palliative care" OR "children hospice" OR "perinatal palliative care")) |  |
| Web of Science | TS=((child* OR adolescen* OR teen* OR youth OR "minor*")  AND (parent* OR mother* OR father* OR caregiver* OR famil*)  AND (cancer OR neoplasm* OR oncolog* OR "palliative care" OR "terminal care")  AND (intervention* OR program* OR counsel* OR psychotherapy OR psychosocial OR psychoeducation OR support* OR therapy))  AND TI=("systematic review" OR "meta-analysis")  NOT TS=("pediatric cancer" OR "childhood cancer" OR "adolescent cancer" OR "pediatric palliative care" OR "children hospice" OR "perinatal palliative care") | 1990/01/01 – 2025/09/01; English, Portuguese, Spanish  Search date: 02/09/2025 |
| SCOPUS | ( TITLE-ABS-KEY ( child* OR adolescen* OR teen* OR youth OR "minor*" ) AND TITLE-ABS-KEY ( parent* OR mother* OR father* OR caregiver* OR famil* ) AND TITLE-ABS-KEY ( cancer OR neoplasm* OR oncolog* OR "palliative care" OR "terminal care" ) AND TITLE-ABS-KEY ( support OR intervention* OR program* OR counsel* OR psychotherapy OR psychosocial OR psychoeducation OR therapy ) ) AND TITLE-ABS-KEY ( "systematic review" OR "meta-analysis" ) AND NOT TITLE-ABS-KEY ( "pediatric cancer" OR "childhood cancer" OR "adolescent cancer" OR "pediatric palliative care" OR "children hospice" OR "perinatal palliative care" ) AND PUBYEAR > 1989 AND ( LIMIT-TO ( DOCTYPE , "re" ) OR LIMIT-TO ( DOCTYPE , "Systematic Review" ) ) AND ( LIMIT-TO ( LANGUAGE , "English" ) ) | 1994/01/01 – 2025/09/02; English, Portuguese, Spanish  Search date: 02/09/2025 |
| APA PsycInfo and PsycArticles (EBSCOhost) | ((SU(Children OR Adolescents OR Minors OR Offspring) OR AB(child* OR adolescen* OR teen* OR youth OR "minor*")) AND (SU(Parents OR Family OR Caregivers) OR AB(parent* OR mother* OR father* OR caregiver* OR famil*)) AND (SU(Neoplasms OR Cancer OR Oncology OR "Palliative Care" OR "Terminal Illness")) AND (SU("Psychosocial Interventions" OR Psychotherapy OR Psychoeducation OR Counseling) OR AB(psychosocial OR psychoeducation OR counseling OR psychotherapy OR intervention* OR program* OR support* OR therapy)) ) AND (PT("Systematic Review") OR PT("Meta Analysis") OR TI("systematic review" OR "meta-analysis")) NOT SU("Pediatric Cancer" OR "Childhood Cancer" OR "Adolescent Cancer" OR "Pediatric Palliative Care") | From the beginning - 2025/09/02; English, Portuguese, Spanish  Search date: 02/09/2025 |
| Epistemonikos | (title:(child* OR preschool* OR toddler* OR infant* OR kid OR "dependent children" OR young OR adolesce* OR teen* OR ("secondary students" OR youth OR "minor children" OR childhood OR son OR daughter OR tween*)) OR abstract:(child* OR preschool* OR toddler* OR infant* OR kid OR "dependent children" OR young OR adolesce* OR teen* OR ("secondary students" OR youth OR "minor children" OR childhood OR son OR daughter OR tween*))) OR (title:((parent* OR mother* OR father* OR caregiver* OR caretaker* OR famil*)) OR abstract:((parent* OR mother* OR father* OR caregiver* OR caretaker* OR famil*))) AND (title:((neoplasm OR cancer OR oncology OR "palliative care" OR "terminal care")) OR abstract:((neoplasm OR cancer OR oncology OR "palliative care" OR "terminal care"))) AND (title:((support OR intervention* OR strateg* OR "best practices" OR treatment OR therapy OR program OR counseling OR psychotherapy OR psychosocial OR psychoeducation OR psychology OR grie* OR bereav* OR mourn* OR effectiveness OR efficacy)) OR abstract:((support OR intervention* OR strateg* OR "best practices" OR treatment OR therapy OR program OR counseling OR psychotherapy OR psychosocial OR psychoeducation OR psychology OR grie* OR bereav* OR mourn* OR effectiveness OR efficacy))) AND title:(("systematic review" OR "meta-analysis")) NOT (title:(("pediatric cancer" OR "childhood cancer" OR "adolescent cancer" OR "pediatric palliative care" OR "children hospice" OR "perinatal palliative care")) OR abstract:(("pediatric cancer" OR "childhood cancer" OR "adolescent cancer" OR "pediatric palliative care" OR "children hospice" OR "perinatal palliative care"))) | 1990/01/01 – 2025/09/03; English, Portuguese, Spanish  Search date: 03/09/2025 |
| Cochrane Database of Systematic Reviews | child* OR preschool* OR toddler* OR infant* OR kid OR dependent NEXT child* OR adolescen* OR teen* OR youth OR minor NEXT child* OR childhood OR secondary NEXT student* OR tween* OR parent* OR mother* OR father* OR caregiver* OR caretaker* OR famil*) in Title Abstract Keyword AND (cancer OR neoplasm* OR oncolog* OR palliative NEXT care OR terminal NEXT care) in Title Abstract Keyword AND (support OR intervention* OR strateg* OR best NEXT practice* OR treatment OR therapy OR program OR counseling OR counselling OR psychotherapy OR psychosocial OR psychoeducation OR psychology OR grie* OR bereav* OR mourn* OR effectiveness OR efficacy) in Title Abstract Keyword AND (systematic NEXT review OR meta-analysis) in Title Abstract Keyword NOT (pediatric NEXT cancer OR childhood NEXT cancer OR adolescent NEXT cancer OR pediatric NEXT palliative NEXT care OR children NEXT hospice OR perinatal NEXT palliative NEXT care) in Title Abstract Keyword - (Word variations have been searched) | 1990/01/01 – 2025/09/03; English, Portuguese, Spanish  Search date: 03/09/2025 |
| ProQuest Dissertations | ("systematic review" OR "meta-analysis")  AND ("parent with cancer" OR "mother with cancer" OR "father with cancer" OR "parental cancer" OR "parental neoplasm*")  AND ("child*" OR "offspring" OR "minor*")  AND (psychosocial OR psychoeducat* OR counsel* OR psychotherap* OR intervention* OR program* OR support* OR therap*)  NOT ("pediatric cancer*" OR "childhood cancer*" OR "childhood malignanc*" OR "cancer in child*" OR "adolescent cancer*" OR "pediatric oncology*" OR "childhood oncology*" OR "pediatric palliative care*") | Only Dissertation/Thesis OR Report OR Conference OR Conference Proceedings; 1990/01/01- 2025/09/07; English, Portuguese, Spanish  Search date: 07/09/2025 |
