# Supplementary material for: Psychosocial interventions for families with minor children affected by parental cancer: An umbrella review
Source: Support Care Cancer. 2026 Jul 11;34(8):756. doi: 10.1007/s00520-026-10999-y (PMC13356062; doi:10.1007/s00520-026-10999-y)
Supplement: Supplementary file 2 — (DOCX 24.3 KB) [file 520_2026_10999_MOESM2_ESM.docx]

**Article title:**
Psychosocial interventions focused on families with minor children affected by parental cancer: An umbrella review

**Journal:**
Supportive Care in Cancer

**Authors:**
Sofia Santos, Raquel Ribeiro, Miguel Barbosa

**Corresponding author:**
Sofia Santos

Faculty of Psychology, University of Lisbon. Lisbon. Portugal

anassantos2@edu.ulisboa.pt

**Online Resource 2**

Excluded studies (n = 37) and justification

| Excluded studies | Justification |
| --- | --- |
| Ahn, S., Romo, R. D., & Campbell, C. L. (2020). A systematic review of interventions for family caregivers who care for patients with advanced cancer at home. *Patient Education and Counseling*, *103*(8), 1518–1530. <https://doi.org/10.1016/j.pec.2020.03.012> | Not parental cancer/minor children specific |
| Applebaum, A. J., & Breitbart, W. (2013). Care for the cancer caregiver: A systematic review. *Palliative and Supportive Care*, *11*(3), 231–252. <https://doi.org/10.1017/s1478951512000594> | Not parental cancer/minor children specific |
| Badr, H., Carmack, C. L., & Diefenbach, M. A. (2015). Psychosocial Interventions for Patients and Caregivers in the Age of New Communication Technologies: Opportunities and Challenges in Cancer Care. *Journal of Health Communication*, *20*(3), 328–342. <https://doi.org/10.1080/10810730.2014.965369> | Not parental cancer/minor children specific |
| Becqué, Y. N., van der Wel, M., Aktan‐Arslan, M., Driel, A. G. van, Rietjens, J. A. C., van der Heide, A., & Witkamp, E. (2023). Supportive interventions for family caregivers of patients with advanced cancer: A systematic review. *Psycho-Oncology*, *32*(5), 663-681. <https://doi.org/10.1002/pon.6126> | Not parental cancer/minor children specific |
| Bilgin, A., & Ozdemir, L. (2021). Interventions to Improve the Preparedness to Care for Family Caregivers of Cancer Patients. *Cancer Nursing*, *45*(3). <https://doi.org/10.1097/ncc.0000000000001014> | Not parental cancer/minor children specific |
| Cheng, Q., Xu, B., Ng, M. S. N., Duan, Y., & So, W. K. W. (2022). Effectiveness of psychoeducational interventions among caregivers of patients with cancer: A systematic review and meta-analysis. *International Journal of Nursing Studies*, *127*(104162), 104162. <https://doi.org/10.1016/j.ijnurstu.2021.104162> | Not parental cancer/minor children specific |
| Chi, N.-C., Demiris, G., Lewis, F. M., Walker, A. J., & Langer, S. L. (2016). Behavioral and Educational Interventions to Support Family Caregivers in End-of-Life Care: A Systematic Review. *The American Journal of Hospice & Palliative Care*, *33*(9), 894–908. <https://doi.org/10.1177/1049909115593938> | Not parental cancer/minor children specific |
| Christodoulou, L., Parpottas, P., & Petkari, E. (2024). Psychological interventions to enhance positive outcomes in adult cancer caregivers: A systematic review. *Journal of Health Psychology*, 29(7), 747-769. <https://doi.org/10.1177/13591053241236254> | No full text |
| Fu, F., Zhao, H., Tong, F., & Chi, I. (2017). A Systematic Review of Psychosocial Interventions to Cancer Caregivers. *Frontiers in Psychology*, *8*. <https://doi.org/10.3389/fpsyg.2017.00834> | Not parental cancer/minor children specific |
| Gabriel, I., Creedy, D., & Coyne, E. (2020). A systematic review of psychosocial interventions to improve quality of life of people with cancer and their family caregivers. *Nursing Open*, *7*(5), 1299-1312. <https://doi.org/10.1002/nop2.543> | Not parental cancer/minor children specific |
| Galway, K., Black, A., Cantwell, M., Cardwell, C. R., Mills, M., & Donnelly, M. (2012). Psychosocial interventions to improve quality of life and emotional wellbeing for recently diagnosed cancer patients. *Cochrane Database of Systematic Reviews*, *11*. <https://doi.org/10.1002/14651858.cd007064.pub2> | Not parental cancer/minor children specific |
| Guan, T., Cook, P., Xu, S., Ranzinger, L. H., Conklin, J. L., Alfahad, A. A.S., Ping, Y., Shieh, K., Barroso, S., Villegas, N., & Song, L. (2023). Family-based psychosocial interventions for adult Latino patients with cancer and their caregivers: A systematic review. *Frontiers in Psychology*, *14*. <https://doi.org/10.3389/fpsyg.2023.1052229> | Not parental cancer/minor children specific |
| Guan, T., Qan'ir, Y., Conklin, J. L., Zimba, C. C., Bula, A., Jumbo, W., Wella, K., Mapulanga, P., Bingo, S. A. M., Chilemba, E., Haley, J., Montano, N. P., Bryant, A. L., & Song, L. (2023). Systematic review of psychosocial interventions for adult cancer patients and their family caregivers in Sub-Saharan Africa. *Global Public Health*, *18*(1), 2199062. <https://doi.org/10.1080/17441692.2023.2199062> | Not parental cancer/minor children specific |
| Harding, R., & Higginson, I. J. (2003). What is the best way to help caregivers in cancer and palliative care? A systematic literature review of interventions and their effectiveness. *Palliative Medicine*, *17*(1), 63–74. <https://doi.org/10.1191/0269216303pm667oa> | Not parental cancer/minor children specific |
| Harding, R., List, S., Epiphaniou, E., & Jones, H. (2012). How can informal caregivers in cancer and palliative care be supported? An updated systematic literature review of interventions and their effectiveness. *Palliative Medicine*, *26*(1), 7–22. <https://doi.org/10.1177/0269216311409613> | Not parental cancer/minor children specific |
| Hartmann, M., Bäzner, E., Wild, B., Eisler, I., & Herzog, W. (2010). Effects of Interventions Involving the Family in the Treatment of Adult Patients with Chronic Physical Diseases: A Meta-Analysis. *Psychotherapy and Psychosomatics*, *79*(3), 136–148. <https://doi.org/10.1159/000286958> | Not parental cancer/minor children specific |
| Hudson, P. L., Remedios, C., & Thomas, K. (2010). A systematic review of psychosocial interventions for family carers of palliative care patients. *BMC Palliative Care*, *9*(1), 17. <https://doi.org/10.1186/1472-684x-9-17> | Not parental cancer/minor children specific |
| Hughes, M. C., Afrin, S., & Hamlish, T. (2023). Effectiveness of Skill-Building Interventions for Informal Caregivers of Adults with Cancer: a Systematic Review. *Journal of Cancer Education*, *38*(2), 390–397. <https://doi.org/10.1007/s13187-022-02236-x> | Not parental cancer/minor children specific |
| Ing, V., Patterson, P., Szabo, M., & Allison, K. R. (2019). Interventions available to adolescents and young adults bereaved by familial cancer: a systematic literature review. *BMJ Supportive & Palliative Care*, *12(e5),* e632-e640 . <https://doi.org/10.1136/bmjspcare-2019-001959> | Not parental cancer/minor children specific |
| Kalyani, C. V., Rohilla, K. K., Gupta, P., Gupta, A., & Gupta, S. (2023). Effect of Psychosocial Interventions on Cancer’s Caregiver Quality of Life: Meta-analysis. *Clinical Practice & Epidemiology in Mental Health*, *19*(1), e 174501792308240. <https://doi.org/10.2174/17450179-v19-e230927-2022-ht14-4336-1> | Not parental cancer/minor children specific |
| Kao, C., Wang, D., Pan, W., Hou, L., Zhou, P., Zhang, Z., Yu, L., Wang, F., & Liu, L. (2025). The efficacy of psychosocial interventions on anxiety and depression in cancer caregivers: a network meta-analysis. *Supportive Care in Cancer*, *33*(6), 513. <https://doi.org/10.1007/s00520-025-09554-y> | Not parental cancer/minor children specific |
| Kühne, F., Krattenmacher, T., Beierlein, V., Grimm, J. C., Bergelt, C., Romer, G., & Möller, B. (2012). Minor Children of Palliative Patients: A Systematic Review of Psychosocial Family Interventions. *Journal of Palliative Medicine*, *15*(8), 931–945. <https://doi.org/10.1089/jpm.2011.0380> | Not cancer specific |
| Kusi, G., Atenafu, E. G., Boamah Mensah, A. B., Lee, C. T., Viswabandya, A., Puts, M., & Mayo, S. (2022). The effectiveness of psychoeducational interventions on caregiver‐oriented outcomes in caregivers of adult cancer patients: A systematic review and meta‐analysis. *Psycho-Oncology*, *32*(2), 189-202. <https://doi.org/10.1002/pon.6050> | Not parental cancer/minor children specific |
| Landi, G Landi, G., Pakenham, K. I., Bao, Z., Cattivelli, R., Crocetti, E., Tossani, E., & Grandi, S. (2025). Efficacy of psychosocial interventions for young offspring of parents with a serious physical or mental illness: Systematic review and meta-analysis. *Clinical Psychology Review*, 118, 102569. <https://doi.org/10.1016/j.cpr.2025.102569> | <50% of the sample met inclusion criteria |
| Lee, J. Z. J., Chen, H. C., Lee, J. X., & Klainin-Yobas, P. (2021). Effects of psychosocial interventions on psychological outcomes among caregivers of advanced cancer patients: a systematic review and meta-analysis. *Supportive Care in Cancer*, *29*(12), 7237–7248. <https://doi.org/10.1007/s00520-021-06102-2> | Not parental cancer/minor children specific |
| Low, N. J. H., Leow, D. G. W., & Klainin-Yobas, P. (2024). Effectiveness of Technology-Based Psychosocial Interventions on Psychological Outcomes Among Adult Cancer Patients and Caregivers: A Systematic Review and Meta-Analysis. *Seminars in Oncology Nursing, Metastatic Breast Cancer Survivorship*, *40*(1), 151533. <https://doi.org/10.1016/j.soncn.2023.151533> | Not parental cancer/minor children specific |
| Northouse, L. L., Katapodi, M. C., Song, L., Zhang, L., & Mood, D. W. (2010). Interventions with family caregivers of cancer patients: Meta-Analysis of randomized trials. *CA: A Cancer Journal for Clinicians*, *60*(5), 317–339. <https://doi.org/10.3322/caac.20081> | Not parental cancer/minor children specific |
| Ohan, J. L., Jackson, H. M., Bay, S., Morris, J. N., & Martini, A. (2020). How psychosocial interventions meet the needs of children of parents with cancer: A review and critical evaluation. *European Journal of Cancer Care*, *29*(5), e13237. <https://doi.org/10.1111/ecc.13237> | A review and critical evaluation (not systematic review) |
| Preyde, M., & Synnott, E. (2009). Psychosocial Intervention for Adults With Cancer: A Meta-Analysis. *Journal of Evidence-Based Social Work*, *6*(4), 321–347. <https://doi.org/10.1080/15433710903126521> | Not parental cancer/minor children specific |
| Sak‐Dankosky, N., Sherwood, P., Vehviläinen‐Julkunen, K., & Kvist, T. (2022). Interventions improving well‐being of adult cancer patients’ caregivers: A systematic review. *Journal of Advanced Nursing*, *78*(9), 2747–2764. <https://doi.org/10.1111/jan.15320> | Not parental cancer/minor children specific |
| Secinti, E., Fischer, I. C., Brennan, E. A., Christon, L., & Balliet, W. (2023). The efficacy of psychosocial interventions for cancer caregiver burden: A systematic review and meta-analysis of randomized controlled trials. *Clinical Psychology Review*, *99*, 102237. <https://doi.org/10.1016/j.cpr.2022.102237> | Not parental cancer/minor children specific |
| Soikkeli-Jalonen, A., Mishina, K., Virtanen, H., Charalambous, A., & Haavisto, E. (2021). Supportive interventions for family members of very seriously ill patients in inpatient care: A systematic review. *Journal of Clinical Nursing*, *30*(15-16), 2179–2201. <https://doi.org/10.1111/jocn.15725> | <50% of the sample met inclusion criteria |
| Treanor, C. J., Santin, O., Prue, G., Coleman, H., Cardwell, C. R., O’Halloran, P., & Donnelly, M. (2019). Psychosocial interventions for informal caregivers of people living with cancer. *Cochrane Database of Systematic Reviews*. <https://doi.org/10.1002/14651858.cd009912.pub2> | Not parental cancer/minor children specific |
| Ussher, J. M., Perz, J., Hawkins, Y., & Brack, M. (2009). Evaluating the efficacy of psycho-social interventions for informal carers of cancer patients: a systematic review of the research literature. *Health Psychology Review*, *3*(1), 85–107. <https://doi.org/10.1080/17437190903033401> | Not parental cancer/minor children specific |
| Waldron, E. A., Janke, E. A., Bechtel, C. F., Ramirez, M., & Cohen, A. (2013). A systematic review of psychosocial interventions to improve cancer caregiver quality of life. *Psycho-Oncology*, *22*(6), 1200–1207. <https://doi.org/10.1002/pon.3118>  Yıldız, M., Terzioğlu, C., & Ayhan, F. (2024). Psychosocial interventions aimed at family members caring for patients with cancer in the palliative period: A systematic review. *International Journal of Nursing Knowledge*, *35*(2), 136–151. <https://doi.org/10.1111/2047-3095.12423> | Not parental cancer/minor children specific  Not parental cancer/minor children specific |
| Zhang, Y., Flannery, M., Zhang, Z., Underhill-Blazey, M., Bobry, M., Leblanc, N., Rodriguez, D., & Zhang, C. (2024). Digital Health Psychosocial Intervention in Adult Patients With Cancer and Their Families: Systematic Review and Meta-Analysis. *JMIR Cancer*, *10*, e46116–e46116. <https://doi.org/10.2196/46116> | Not parental cancer/minor children specific |
