# Supplementary material for: Psychosocial interventions for families with minor children affected by parental cancer: An umbrella review
Source: Support Care Cancer. 2026 Jul 11;34(8):756. doi: 10.1007/s00520-026-10999-y (PMC13356062; doi:10.1007/s00520-026-10999-y)
Supplement: Supplementary file 3 — (DOCX 33.2 KB) [file 520_2026_10999_MOESM3_ESM.docx]

**Article title:**
Psychosocial interventions focused on families with minor children affected by parental cancer: An umbrella review

**Journal:**
Supportive Care in Cancer

**Authors:**
Sofia Santos, Raquel Ribeiro, Miguel Barbosa

**Corresponding author:**
Sofia Santos

Faculty of Psychology, University of Lisbon. Lisbon. Portugal

anassantos2@edu.ulisboa.pt

**Online Resource 3**

Characteristics of systematic reviews

| Study details | Included studies (n = 8) | | | | | | | |
| --- | --- | --- | --- | --- | --- | --- | --- | --- |
| Author/Year | Alexander et al. (2019) | Chong et al. (2024) | Ellis et al. (2017) | Geertz et al. (2023) | Inhestern et al. (2016) | Malinowski et al. (2025) | Strandh et al. (2023) | Zhao et al. (2024) |
| Objectives | To identify current interventions to support cancer patients’ children and summarize how effective these are based on children’s reports. | To assess the effectiveness of psychosocial interventions targeting parents with cancer on their mental health, quality of life, their children’s well-being,  and family functioning. | To summarize the psychosocial needs of children and adolescents with a parent with cancer across the illness trajectory and evaluate existing  interventions for this population. | To summarize peer-group interventions for children and adolescents of cancer patients. | To provide an overview of existing interventions and support programs and barriers and facilitators for using psychosocial  interventions for families affected by parental cancer. | To summarize  empirical studies of interventions for minor children focusing on child’s  psychological functioning across the parental cancer trajectory. | To describe available psychosocial interventions  for parents with cancer and dependent children. | To summarize the characteristics and effectiveness of interventions aimed at improving  illness-related communication between parents with cancer and their minor children. |
| Participants (characteristics/total number) | **N ≈** 298 children and adolescents (aged 6–20 years) from parent-child dyads and families;  **Type/Stage of Parent Cancer:** any type, mostly in stages I–III (study focused only on mothers with stage 0–III breast cancer);  **Time since diagnosis:** <12 months;  **Ethnic composition:** 2 studies mentioned African American dyads; | **N =** 496 parents (mean age range = 41.1 to 46.4) with cancer and 558 children (mean age range = 8.2 to 16). Most were mothers with cancer;  **Type/Stage of Parent Cancer:** Breast cancer predominance (31.0%–82.0% of the sample per study). Most studies had parents diagnosed with cancer at stages 0-III, with only one study focusing exclusively on parents with stage IV cancer;  **Time since diagnosis:** mean ranged from 3.7 months to 48.9 months;  **Ethnic composition:** ethnically diverse samples, 2 studies only recruited Hispanic and African American participants; | Total number of participants not mentioned. Mostly school age children (4–11 years) and adolescents (12–18 years), while pre-school age children (1–3 years) were only included in 1 study.  **Type/Stage of Parent Cancer:** most interventions included children of parents with mixed/unspecified cancer diagnoses at various stages of their illness; 2 interventions targeted children of parents with breast cancer, 2 interventions included children of parents with advanced cancer or who had died.  **Time since diagnosis:** ranged from newly diagnosed to the past 6-12 months.  **Ethnic composition:** not mentioned. | Total number of children, adolescents and young adults participants not mentioned, yet there’s more female than male. 78 mothers (76,47%), 20 fathers (19,61%) and 4 other family members or caregiver (3,92%) participated.  **Type/Stage of Parent Cancer:** Mainly breast cancer patients. Studies reported varied cancer stages, from I to III, to remission and only a few were either terminal, deceased or unknown.  **Time since diagnosis:** within 3 to 12 months.  **Ethnic composition:** not mentioned. | Total number of participants not specified. The target group was mostly families with school-aged children.  **Type/Stage of Parent Cancer:** Mostly any kind of parental cancer. Two interventions included only breast cancer patients. The cancer stage ranged from stage I, II, III to incurable or terminal cancer.  **Time since diagnosis:** not mentioned.    **Ethnic composition:** participants were from western countries. One study mentioned African American families. | SR 1:  **N =** 534 children aged 4-18 years. The parents ranged in age from 32–51 with most parents being in their 40s.  **Type/Stage of Parent Cancer:** most cancer parents were mothers, with breast cancer being the most common cancer. Stage ranged from early to advanced metastatic cancer.    **Time since diagnosis:** when mentioned, ranged from within 6 months to 5 years.  **Ethnic composition:** nor mentioned.  SR 2:  **N =** 20 bereaved children, aged 7–12 years. Most children (95%) had lost their parent within the 12 months prior to the program. 45% percent had lost their mother and 55% had lost their father.  **Type/Stage/Time since diagnosis of Parent Cancer:** not mentioned.  **Ethnic composition:** 70% Chinese children. | **N =** 1646 participants in the interventions (including 41% children, n = 683, and 13% spouses or other relatives, n = 207). Ages of the children ranged from 3-17 years.  **Type/Stage of Parent Cancer:** various cancer types, from non-metastatic to advanced cancer.  **Time since diagnosis:** not mentioned.  **Ethnic composition:** not mentioned. | **N =** 213 families, 149 parents, and 192 minor children (mostly under 18) was included. The average age ranged from 34 to 56 years. More females than males among parents and minor children.  **Type/Stage of Parent Cancer:** various cancer types, ranged from 0-III to palliative stage of cancer.  **Time since diagnosis:** not mentioned.  **Ethnic composition:** not mentioned. |
| Setting/context | Participants were from any type of settings including community,  hospital or residential. Most interventions were provided at home or in a clinical setting such as a hospital. | Participants recruitment from any setting. Interventions were delivered at patient's home or elsewhere. | Interventions occurred in home-based, school-based and structured  family based counselling. | Peer-group setting; varied intervention setting (e.g., adventure camp or weekly groups). | Different intervention's setting, including group format with several families, children or parents or meetings with single families or family member), from initial diagnostic phase to sessions before and after death. | SR 1: interventions were delivered in several formats, including individual family, group, in‐person, and virtual interventions. SR 2: in-person group intervention for children. | Interventions were made available individually, to the family, in groups or as a combination of these. The interventions were delivered in different settings: face-to-face, online, via telephone, text material, or a combination. Physical settings also varied where some were offered in the hospital, the parent’s homes or a combination. | Various interventions delivery settings such as hospitals or clinics, palliative care setting, participants' homes, offices or local  schools, web-based and telephone-based interventions. |
| Interventions/Phenomena of interest | Psychosocial interventions that supports patients’ children’s psychological, emotional and behavioural issues. | Psychosocial interventions | Psychosocial interventions | Psychosocial-peer group interventions that includes group sessions with peers’ approach for children and adolescents with a parent who has cancer or who has died of cancer. | Structured psychosocial interventions for families with minor children affected by parental cancer focused on treatment or prevention of negative psychosocial consequences, on single family members (children, parents, spouses) or the whole family. | Interventions for children when a parent has cancer and bereavement interventions for children when a parent dies. | Psychosocial interventions targeting the ill parent’s mental health and parenting distress following any type of cancer in parents of children under the age of 18, as well as intervention outcomes for the ill parent. | Any intervention aimed at improving communication between parents with cancer and their minor children about the diagnosis. |
| Sourced searched | Between 25 May 2015 and 6 August 2018, Medline, PsycInfo, ProQuest, Cochrane,  CINAHL, Embase and Google Scholar databases were searched. Grey literature was searched in Google, ProQuest conferences and Proquest theses. Hand-searched journals included Journal of Psychosocial  Oncology, Psycho-oncology and Cancer. The reference lists of identified studies were also searched. | From inception to 7th July 2023, Ovid MEDLINE, PubMed, PsycINFO, and Cochrane Central Register of Controlled Trials databases were searched. | From 1985 to 2015, Medline, CINAHL, PsychInfo, EMBASE and Social Work Abstracts were searched. | In May 2021 with an update in February 2022, PsycINFO, Web of Science, MEDLINE, and CINAHL were searched. No date restriction was applied. The reference lists of identified articles were also searched. | In June 2014, with an updated in September 2015, EMBASE, MEDLINE, PsycInfo, Psyndex, CINAHL) were searched. | Ovid Medline, PsycINFO, Cumulative Index to Nursing and Allied Health Literature (CINAHL), Sociological  Abstracts, and Social Services Abstracts were searched for articles published in 2015 and beyond. | A first search for studies published between January 2000 and December  2021 was made on PubMed, PsychINFO, CINAHL and Web. The search was updated in May 2022 and March 2023. | CINAHL, Web of Science, PubMed, Embase, the Cochrane Library, and PsycINFO were searched for articles published between 2000 and 2023. |
| Range (years) of included studies | 2006-2015 | 2006-2023 | 1992-2015 | 2013-2021 | 1990-2015 | 2015-2023 | 2003-2023 | 2006-2022 |
| Number of studies included | 8 | 11 | 12 reported on children’s psychosocial needs + 12 intervention studies (only intervention studies were included) | 10 | 36 (of these 19 are intervention studies) | SR 1: 11, SR2: 1 | 30 | 21 |
| Type of studies included | Quantitative (2 RCT, 1 exploratory quasi-experimental,1 quasi-experimental, and 1 pilot-study) and Mixed-methods (1 controlled experimental design, no randomization, 1 quasi-RCT and 1 pilot controlled trial) studies. | Quantitative (4 studies adopted one-group pretest-posttest design, 4 studies adopted two-group pretest posttest design, and 3 studies adopted RCT design). | 8 qualitative studies, 2 RCT and 3 NRCT. | Quantitative (2-year not-randomized quasi-experimental two-group pilot study, pre- and post-design; not-randomized three-arm pre-post design: children’s group, parents seminar and waitlist control group; pilot intervention study, within-subject-control-group design; quasi- experimental within-subject design comparing pre- and post-test assessments of children and parents in separate analyses; observational prospective study, pre-post and 6 month follow-up), qualitative (focus group; descriptive qualitative design) and Mixed-methods (Multisite quantitative and qualitative evaluation, pre-post-design; quantitative questionnaire pre-post design and qualitative 3-month-follow up interview) studies. | 16 were evaluation studies (n = 6 qualitative, n = 9 quantitative, n = 1 mixed methods), whereas 20 articles focused on the development  or feasibility and implementation of interventions. | SR1: 4 RTCs and 7 single group, pre and post‐intervention quasi‐experimental design. SR 2: Non‐randomized, pre‐test–posttest pilot study. | 6 (RCTs), however, 3 of these were conducted as non-randomized pre- /post intervention studies; 13 planned and conducted pre-/post intervention studies were included, as were 8 qualitative studies and 3 study protocols. | 4 RCTs and 17 quasi-experimental studies. |
| Country of origin of included studies | Iran (n = 1), USA (n = 4), Norway (n = 1), Japan (n = 1) and Germany (n = 1) | USA (n = 8), Norway (n = 1), Finland (n = 1) and Germany (n = 1). | USA (n = 6), Norway (n = 1), Canada (n = 1), Germany (n = 1), Malta (n = 1), Ireland (n = 1) and Denmark (n = 1). | USA (n = 3), Germany (n = 2), Ireland (n = 2), Sweden (n = 1), Australia (n = 1) and Japan (n = 1). | USA (n = 16), Germany (n = 10), Norway (n = 2), Australia (n = 2), Finland (n = 2), Canada (n = 1), Denmark (n = 1), UK (n = 1) and Israel (n = 1). | SR 1: Switzerland (n = 1), Norway (n = 1), Iran (n = 1), Japan (n = 1), and the USA (n = 7). SR 2: Singapore (n = 1). | USA (n = 13), Germany (n = 4), Australia (n = 3), Switzerland (n = 2), UK (n = 2), Denmark  (n = 1), Finland (n = 1), Israel (n = 1), Japan (n = 1), Norway (n = 1) and Sweden (n = 1). | Germany (n = 4), USA (n = 7), Norway (n = 2), Australia (n = 2), Israel (n = 1), Switzerland (n = 2) and Sweden (n = 3). |
| Appraisal instruments used | Keim-Malpass and Colleagues appraisal tool. Only the quantitative component of the mixed design studies was  assessed. | Quality Assessment Tool for Quantitative Studies by Effective Public Health Practice Project (EPHPP) | Mixed Method Appraisal Tool (MMAT) | Mixed Method Appraisal Tool (MMAT) | Mixed Method Appraisal Tool (MMAT) | Joanna Briggs Institute Critical Appraisal Checklist for Studies Reporting  Prevalence Data | Mixed Method Appraisal Tool (MMAT) | Joanna Briggs Institute Critical Appraisal Checklist for RCTs and quasi-experimental studies |
| Appraisal rating | Overall, the methodological quality of studies in was not high. | The overall rating assigned to most studies was weak, with only two rated as moderate. | Methodological quality ranged from low to high across the qualitative studies (n = 11), with 3 non-randomized controlled trials intervention studies rated between moderate and high quality (including one mixed methods study). | Methodological quality ranged from low to adequate. | Overall methodological quality rated as moderate. | Overall, quality  assessment indicated a generally low risk of bias and high methodological quality for both reviews. | The study quality was, overall, assessed as moderate. | The quality of the included studies ranged from 6 to 9 on standardized assessment scales. |
| Method of analysis | Narrative synthesis. Only the quantitative components of the mixed methods studies have been presented. | Narrative synthesis. | Narrative synthesis. | Narrative synthesis. | Narrative analysis to synthesize the data and thematic synthesis to identify barriers and facilitators for using psychosocial intervention. | Not mentioned in the study. Compatible with narrative synthesis. | Narrative analysis  and synthesis. | Narrative synthesis. |
| Outcomes assessed | Change or no change reported by children on psychological, emotional and behavioural issues (e.g., PTSD symptoms, emotional regulation, depression and anxiety). | Primary outcomes: mental health outcomes of parents diagnosed with cancer (e.g., anxiety, depression, parenting stress and/or psychological distress in general);  Secondary outcomes: mental health of children (e.g., anxiety, depression, internalizing problems, externalizing problems, anxious/ depressed mood), the quality of life of both ill parents and their children and family functioning and quality of life within the parent-child dyads. | Child/adolescent outcomes (e.g. intervention satisfaction, communication, knowledge, psychological and family functioning). Outcomes could be assessed by qualitative and/or quantitative measures completed by the child/adolescent, parent or relevant health professional/intervention facilitator. | Any psychosocial or intervention evaluation outcome, quantitatively evaluated and/or qualitatively reported (e.g.feasibility, acceptance, participants` general feedback and evaluation of  psychosocial effects of the intervention). | Family (e.g., quality of life of parents and children, distress and mental health, family functioning, communication, sharing, reassurance and normalization of emotions, coping strategies); parent-centred (e.g., improvement in parent’s functioning, parenting skills, emotional and behavioural problems, quality of mother-child relationship and communication competence) and child-centred (social bond between the children, deal with difficult situations and master challenges, understanding of cancer, coping strategies, mood and behaviour of the children) outcomes. | Only child outcomes (e.g., quality of life, family functioning, behavioral and emotional functioning,  anxiety, stress, post‐traumatic stress, and worries about the  parent's illness), either reported by parent and/or child. | Parental outcomes (e.g., improve the ill parent’s mental health and reduce parenting distress). | Parents and/or minor children's  knowledge and understanding about cancer and its treatment  or psychological, social, and behavioral outcomes. |
| Results/Findings | Eight studies evaluated six interventions (The Enhancing Connection Program; A Supportive-educative program; A culturally adapted program; Children’s Lives Include Moments of Bravery (CLIMB); Children of Somatically Ill Parents (COSIP) Multinational; and Cancer PEPSONE Program (CPP) that both supported patients’ children and engaged children in the evaluation process.  A limited number of significant results were reported by studies. These evidenced improvement for PTSD symptoms, emotional regulation, and depression, but not for anxiety.  Overall current interventions do not appear effective among patients’ children. | Interventions aimed to strengthen parent-child connection (Enhancing Connection), enhance family communication, improve psychological well-being of parents (Struggle for Life Intervention), and address children’s mental health (Wonders and Worries). Additionally, interventions like Cancer PEPSONE Program (CPP) aimed to expand social networks and support systems.  These interventions demonstrated success in reducing depressive and anxiety symptoms, parenting stress, and mitigating children’s externalizing and internalizing problems.  However, there are methodological limitations such as participant selection bias, lack of blinding, and low follow-up rates. | Twelve interventions developed for this population were identified.  All 8 qualitative studies obtained positive outcomes, particularly in terms of children's knowledge and understanding of parental cancer, emotional management, and family functioning and communication.  Two RCTs showed improvements in depression in both children and parents, as well as in children's behavior and parenting skills. No significant changes to child or maternal anxiety, or parenting self-efficacy. However, when parents were in palliative care had died, there were no improvements in self-esteem, depression, and anxiety, but there was a significant increase in family communication.  In NRCT studies, the outcomes were also positive, highlighting a decrease in emotional distress, improvement in depression, and family communication skills.  Difficulty to identify clear evidence regarding the efficacy of interventions included, due to the large proportion of qualitative intervention studies, lack of controlled data and considerable heterogeneity in outcome measures adopted across studies. | Ten studies evaluated ten different peer-group interventions for children and adolescents with a parent diagnosed with cancer. Overall, participants considered the group intervention to have a positive effect on reducing isolation, normalization among peers, talking safely about emotions, gaining cancer specific knowledge, skills and coping strategies and being able to temporarily forget about worries and to have fun. These aspects improved psychological well-being and quality of life of the children and adolescents.High acceptance and feasibility of peer-group interventions. | Nineteen different interventions for families with minor children affected by parental cancer. 8 interventions focused on parents and children, 7 were children-centred and 4 mainly focused on working with parents.  Results suggest that interventions are helpful and that participants improved in various outcomes. | SR 1: Some of the studies demonstrate efficacy in improving child behavioral and emotional adjustments. Limited literature  on effective interventions for children during their parent's cancer treatment.  SR 2: All participants reported finding the group helpful. The art activities helped them to express their feelings of loss and to feel less alone in their grief. | Thirty studies mapped and described 23 unique psychosocial interventions. In studies evaluating interventions, different aspects of parents’ wellbeing often improved as parenting distress decreased. Interventions were described as helpful by the parents receiving them.  These are preliminary findings due to methodological problems | The interventions were categorized as family-centered, parent-centered, or children-centered. Family-centered interventions showed unique advantages in improving family life; parent-centered interventions brought benefits in enhancing parenting quality, parents' self-efficacy in coping with cancer, and children's social behavior; and child-centered interventions had a significant impact on the psychological well-being of children.  The interventions emphasized disease knowledge, communication skills, emotional management, and future planning in illness-related communication. |
| Significance/Direction | Need for more targeted, child-centred interventions and future validation studies that adhere to the appropriate intervention and methodological guidelines and adequately engage children in the evaluation process.  The development of a theoretical model conceptualizing how children are affected by their parent’s cancer diagnosis would be important. | Absence of robust evidence supporting the definitive effectiveness of interventions in enhancing the evaluated outcomes.  Future research should comprehensively explore the multifaceted needs of family members confronting parental cancer and their quality of life.  The recommendation to conduct rigorous clinical trials to substantiate the evidence. | Knowledge of children’s needs across developmental stages, and at different stages in their parent’s disease is urgently needed, to find out the specific elements of interventions that are likely to yield the largest benefits.  Rigorous and evidence-based interventions to inform clinical practice are needed. | Peer-group interventions are an effective way to provide psychosocial support when a parent has cancer.  More psychosocial interventions (both individual counselling and support groups or family sessions) to address the different needs of all family members should be offered along the course of parental cancer disease. | The development and the implementation of intervention programs for families with minor children and parental cancer needs careful planning of the intervention and the implementation process.  Crucial components of successful implementation involves building tight collaborations as well as anticipating barriers to using the support offers. | Evidence‐based interventions for minor children whose parents have cancer or who are bereaved during childhood are limited.  The authors propose a model to guide the development of interventions for children throughout the illness and bereavement trajectory. | Only a few of developed psychosocial interventions are implemented into cancer care to meet the needs of parents with cancer and their families.  For a successful intervention, researchers and practitioners should consider aspects as being a brief and telephone delivered, and that still builds a relationship with the provider. | To enrich the content of parent-centered interventions, effects of interventions on communication should be directly assessed, and high-quality RCT studies should be conduct in order to provide robust evidence to support these findings.  Educating about the disease, using web-based interventions, and fully considering children's ages may improve illness-related communication between parents with cancer and their minor children in oncology practice. |
| Heterogeneity | High level of heterogeneity across study research designs and interventions. | Presence of clinical and methodological variations. | Variability in study design and quality and considerable heterogeneity in intervention characteristics and outcome variables. | High degree of heterogeneity in the methodology, implementation and focus of the group interventions. | Heterogeneous theoretical background, primary aims and impact of the interventions. | SR 1: Heterogeneous study populations; Interventions were also varied. | Heterogeneity in intervention aim, target group, components, procedure and outcomes. | Significant heterogeneity among the included studies. |
| Comments | Few studies included, high heterogeneity, inconsistent and limited results. Cautious interpretation. | Benefits reported in some relevant outcomes, but evidence limited by high heterogeneity and poor methodological quality. Cautious interpretation. | Preliminary results show benefits; conclusions on effectiveness limited by heterogeneity and low methodological quality. Cautious interpretation. | Promising results, but evidence limited by heterogeneity and methodological limitations. Cautious interpretation. | Promising results, but evidence limited by high heterogeneity and poor methodological quality. Cautious interpretation. | Promising but inconsistent results. Evidence limited by high heterogeneity and poor methodological quality. Cautious interpretation. | Promising results, but limited by the risk of bias. Cautious conclusions due to variable methodological quality. | Promising parent-centered interventions, but mixed/limited effects on parents’ QoL and psychological outcomes. High heterogeneity; no meta-analysis possible. |
