# Supplementary material for: Psychosocial interventions for families with minor children affected by parental cancer: An umbrella review
Source: Support Care Cancer. 2026 Jul 11;34(8):756. doi: 10.1007/s00520-026-10999-y (PMC13356062; doi:10.1007/s00520-026-10999-y)
Supplement: Supplementary file 4 — (DOCX 44.4 KB) [file 520_2026_10999_MOESM4_ESM.docx]

**Article title:**
Psychosocial interventions focused on families with minor children affected by parental cancer: An umbrella review

**Journal:**
Supportive Care in Cancer

**Authors:**
Sofia Santos, Raquel Ribeiro, Miguel Barbosa

**Corresponding author:**
Sofia Santos

Faculty of Psychology, University of Lisbon. Lisbon. Portugal

anassantos2@edu.ulisboa.pt

**Online Resource 4**

Summary of psychosocial interventions (n = 46) characteristics included in systematic reviews (n = 8)

| Name of the intervention | Identified SRs | Stage of parental cancer | Focus of the intervention | Participants/setting | Intervention  theory | Intervention aim | Intervention strategies | Intervention components | Intervention timing | Intervention mode of delivery/setting | Intervention provider |
| --- | --- | --- | --- | --- | --- | --- | --- | --- | --- | --- | --- |
| The Enhancing Connection Program (Lewis 2015; Lewis 2006) | Alexander et al., 2019; Chong et al. 2024; Ellis et al. 2017, Inhestern et al. 2016; Malinowski et al. 2025; Strandh et al. 2023; Zhao et al. 2024 | Stage 0‐III  breast cancer | Il parent-focused | Mothers with breast cancer | Contextual  model of  parenting;  Transtheoretical coping theory;  Social cognitive theory | Reduce cancer-related distress, strengthen parenting  behaviour and  improve children’s  adjustment | Education counselling; Scripted patient education sessions,  child and parent interative workbooks and  phone access to patient educator | Key contents:  1.Anchoring yourself to help your  child  -Details: Manage own emotions  and understand child’s  experience  -Goal: Be an attentive listener to  child  2.Adding to your listening skills  -Details: Develop communication  skills with child  -Goal: Support mothers in  communicating with child  3.Building on your listening skills  -Details: Further develop  communication skills with child  -Goal: Support mothers in  communicating with introvert  child  4.Being a detective of your child’s  coping  -Details: Help mothers  understand child’s coping  behaviours  -Goal: Help mothers respond  positively to child’s coping  5.Celebrating your success  -Details: Reflect on achievements  in the program  -Goal: Internalize a new view of  identity | 5 sessions delivered at 2 week intervals, 60 min per session, across 10 weeks | Face-to-face intervention, home-based sessions or elsewhere participants choose | Trained patient educators  (no information about number and  qualification of educators) |
| Enhancing Connections-Telephone Program (EC-T) (Lewis et al., 2017; Walker et al. 2018) | Chong et al., 2024; Malinowski et al., 2025; Strandh et al. 2023, Zhao et al., 2024 | Stage 0‐III breast  cancer | Ill parent-focused | Mothers with breast cancer | Developmental-contextual model of parenting, the transtheoretical model of coping, and Bandura’s social cognitive theory | Decrease maternal depressed mood and anxiety, improve parenting behaviour (parenting quality, skills and self-efficacy), and improve children’s behavioural-emotional adjustment to their mother’s breast cancer. | Psycho-educative, reflective and skill- and efficacy-building elements | Key contents 1.Anchoring yourself to help your child -Details: Manage own emotions and understand child’s experience -Goal: Be an attentive listener to child 2.Adding to your listening skills -Details: Develop communication skills with child -Goal: Support mothers in communicating with child 3.Building on your listening skills -Details: Further develop communication skills with child -Goal: Support mothers in communicating with introvert child 4.Being a detective of your child’s coping -Details: Help mothers understand child’s coping behaviors -Goal: Help mothers respond positively to child’s coping 5.Celebrating your success -Details: Reflect on achievements in the program -Goal: Internalize a new view of identity | Five 1-h education counselling sessions at 2-week intervals, across 10 weeks | Telephone delivered;  Patient’s home | Trained patient educators (number of educators not provided) with standardized training and weekly follow-up meetings within intervention period |
| Enhancing Connections- Palliative Care Program (ECPC) (Lewis et al., 2020; Zahlis et al. 2020) | Chong et al., 2024; Malinowski et al., 2025, Strandh et al., 2023; Zhao et al., 2024 | Advanced and palliative stage of cancer | Ill-parent focused | Parents with various types of cancer | Trans-theoretical model of coping, contextual model of parenting, Bandura’s social cognitive theory | Decrease maternal depressed mood and anxiety, improve parenting behaviour (parenting quality, skills and self-efficacy), and improve children’s behavioural-emotional adjustment to their mother’s breast cancer | Psycho-educative, reflective and skill- and efficacy-building elements | Key Contents 1.Anchoring yourself to help your child -Details: Manage own emotions and understand child’s experience under parental advanced cancer -Goal: Establish self-care skills in parents 2.Adding to your listening skills -Details: Develop communication skills with child -Goal: Support mothers in communicating with child 3.Building on your listening skills -Details: Help with children elaborating their concerns -Goal: Support mothers in communicating with introvert child 4.Being a detective of your child’s coping -Details: Help mothers understand child’s coping behaviors non-judgmentally -Goal: Help mothers respond positively to child’s coping 5.Celebrating your success -Details: Reflect on achievements in the program -Goal: Internalize a new view of identity | 5 education counselling sessions (1-h) over 10 weeks and home assignments as well as support by the patient educator in between | Telephone-delivered; scripted  treatment  manual | Trained nurse (number of nurses and qualification not provided) |
| Enhancing Connections- Group Program (EC-G) (Lewis et al., 2021) | Chong et al., 2024; Malinowski et al. 2025; Strandh et al., 2023 | Stage 0‐III | Parent-focused | Parent or surrogate  parent (both ill and  well) with a child  5–17 living in the home | Trans-theoretical model of coping, contextual model of parenting, Bandura’s social cognitive theory | Decrease maternal depressed mood and anxiety, improve parenting behaviour (parenting quality, skills and self-efficacy), and improve children’s behavioural-emotional adjustment to their mother’s breast cancer | Psycho-educative, reflective and skill- and efficacy-building elements | 1.Anchoring yourself to help your child -Details: Manage own emotions and understand child’s experience -Goal: Be an attentive listener to child 2.Adding to your listening skills -Details: Develop communication skills with child -Goal: Support mothers in communicating with child 3.Building on your listening skills -Details: Further develop communication skills with child -Goal: Support mothers in communicating with introvert child 4.Being a detective of your child’s coping -Details: Help mothers understand child’s coping behaviors -Goal: Help mothers respond positively to child’s coping 5.Celebrating your success -Details: Reflect on achievements in the program -Goal: Internalize a new view of identity | Five 1-h education counselling sessions at 2-week intervals, across 10 weeks | In‐person  group  -Group-delivered (group size not mentioned);  -Community setting | 6 trained group facilitators with fully scripted and two days of training |
| A Supportive-educative program (Azarbarzin et al., 2015) | Alexander et al., 2019 | Any cancer types | Adolescent focused | Adolescents  with a  parent with  cancer | Not mentioned | Improve child’s QoL | Individually at  home or in  small lecture  groups (varied)  Self-directed | Not mentioned | 45-90 mins per session (depending on needs) | Individual/group  information and  counselling sessions  Booklet, compact disk  and weblog access at  end of program | Not mentioned |
| A culturally adapted program (Davey et al., 2013) | Alexander et al., 2019; Chong et al., 2024; Ellis et al., 2017; Geertz et al., 2023; Inhestern et al., 2016; Strandh et al., 2023; Zhao et al., 2024 | Stage I‐III | Family-focused | African  American families  with school-aged  children | Clarke’s school-age child support group model, Beardslee’s preventive intervention model for families coping with parental cancer  Attachment Theory, Clarke’s school-age child support group model and Beardslee’s preventive intervention model for families coping with parental depression | Improve family communication and parent-child attachment  Improve family communication,  improve parent-child attachment  for African American families  Give culturally sensitive psychosocial support to African-American families with parental cancer | Psycho-educative and discussion elements | Key contents 1.Three sessions (90 min) (To children) -Details: Discuss the feelings and coping strategies among children, improve positive thinking -Goal: Reduce tension, promote family communication 2.Two sessions (2 h) (To parents and children) -Details: Emphasize the importance of African American community, promote open communication in family -Goal: Increase family resilience to parental cancer Intervention modality; frequency and dosage -Children group-based session (at most 5 children per group) + Parents-children group-based session (at most five dyads per group); 5 bimonthly support group sessions, across 10 weeks | 5 sessions over 10 weeks | Face to face;  Support group format (up to 5  children); Manualised treatment | 1 trained African American female therapist with treatment manual and supervision by first author |
| Children’s Lives Include Moments of Bravery (CLIMB) (Kobayashi et al., 2017; Shallcross et al., 2016; Semple and McCaughan, 2013) | Alexander et al., 2019; Ellis et al., 2017; Geertz et al., 2023; Inhestern et al., 2016; Malinowski et al., 2025; Strandh et al., 2023; Zhao et al., 2024 | Cancer diagnosis | Children-focused | School-aged Children with a parent with cancer | Principles of mental health promotion;  Based on the premise that children are powerfully shaped by their external environment and behaviour is a function of social context, which has been referred to as the ‘power of context’;  Banduras social  cognitive theory and  involves evidence-based  approaches including  emotion labelling,  normalization, and  expression | Provide information, normalize emotions improve communication and strengthen connections among children, improve family communication | Psycho-educative and discussion elements | Not mentioned | 6 weeks 1.5 to 2 hours weekly group meetings | In‐person  Group (3-6 participants)  conducted  in university  or  hospital  setting | Psychosocial oncology professionals: social worker, psychologist and child-life specialist |
| Children of Somatically Ill Parents (COSIP) (Thatsum & Munch-Hansen, 2006) | Alexander et al., 2019; Ellis et al., 2017; Inhestern et al., 2016 | Initial diagnostic phase | Family-focused | Well parent  Children  Whole family | Family therapy | Support the family in taking care of the children’s needs as best as possible | Not mentioned | Not mentioned | 5–6 sessions, over an  average of 4.7 months | Focused, short term and needs based  sessions with the whole family at home.  Children were also offered group  counselling with other children in  the project | Not mentioned |
| Cancer PEPSONE Program (CPP) (Hauken et al., 2017) | Alexander et al., 2019; Chong et al., 2024; Malinowski et al., 2025 | Cancer diagnosis  within the last  5 years | Family-focused | Ill parents + Children + Healthy parents | Quality of life theory Learning theory | Investigate intervention effects on children’s anxiety and QoL | Two parts—  psycho‐education  followed by a discussion  between  the family and  their network  members | Key contents 1.Psychoeducation (1 h) -Details: Deliver information about familial cancer, importance of social network during family crisis, support by social network -Goal: Emphasize the importance of social network support 2.Discussing session (1.5 h) -Details: Discuss the needs for social support and support available from social network members -Goal: Provide open communication between family members and social network members | 1 session, 3 hours long | Manual based, face-to-face intervention, at patient’s home or elsewhere they choose | 3 trained clinical psychologists with trainings on standardized intervention manual |
| Let’s Talk about the Children Intervention (LT) (Niemela, 2012) | Chong et al., 2024; Inhestern et al., 2016; Strandh et al., 2023 | Not mentioned | Parent-focused | Ill parents | Not mentioned | Strengthen children | Psycho-education and counselling | Key contents 1.First session -Details: Discuss child’s protective factors -Goal: Induce knowledge to support parenthood 2.Second session -Details: Teach how to strengthen child’s protective factors -Goal: Support children | Face-to-face intervention; 2 sessions; period not mentioned | Not mentioned | Trained counsellor (number of counsellor and qualification not mentioned) |
| Family Talk Intervention (FTI) (Alvariza et al., 2021; Eklund et al., 2022; Niemela et al., 2012) | Chong et al., 2024; Inhestern et al., 2016; Strandh et al., 2023; Zhao et al., 2024 | Not mentioned | Family-focused | Ill parents + Children + Healthy parents | Psycho-education, narrative theory and dialogical theory | Family Talks: support family  communication, support  children’s and parents’  psychosocial well-being | Psycho-educative, reflective and counselling elements | Key contents 1.First and Second session (To parents) -Details: Explore parents’ experience and concerns -Goal: Get a picture of how to support parents 2.Third session (To children) -Details: Explore children’s worries and questions -Goal: Support children 3.Fourth session (To parents) -Details: Plan family discussion -Goal: Facilitate family communication 4.Remaining sessions(s) (To parents and children) -Details: Communicate between parents and children, review the whole program -Goal: Promote family functioning, improve future intervention | Face-to-face intervention; 6 to 8 sessions; period not mentioned | Setting not mentioned | Not mentioned |
| Conexiones (Palacios et al., 2023) | Chong et al., 2024; Malinowski et al., 2025; Strandh et al., 2023 | Stage cancer 0-III | Parent-focused | Ill mothers living in  communities along  the US‐Mexico | Collins’ developmental-contextual model of parenting, Bandura’s Social Cognitive Theory | To culturally adapt Enhancing connections (EC) and decrease maternal depressed mood and anxiety, improve parenting behaviour (parenting quality, skills and self-efficacy), and improve children’s | Psycho-educative, reflective and skill- and efficacy-building elements | Key Content 1.Anchoring yourself to help your child -Details: Manage own emotions and understand child’s experience -Goal: Be an attentive listener to child 2.Adding to your listening skills -Details: Develop communication skills with child -Goal: Support mothers in communicating with the child 3. Checking in with your child about the cancer -Details: Strengthen mothers’ skills in encouraging the child to describe their emotions -Goal: Facilitate mutual communication 5.Celebrating your success -Details: Reflect on achievements in the program -Goal: Build confidence in parenting | 5‐session educational  program over 10 weeks and home assignments as well as support by the patient educator in between | Telephone delivered; at patient’s home or elsewhere they choose | Trained patient educators |
| Wonders and Worries (Phillips et al., 2022) | Chong et al., 2024; Malinowski et al., 2025; Strandh et al., 2023; Zhao et al., 2024 | Stage cancer 0-III | Child-focused | Ill parent, Child and then Parent- Child dyad | Child Life Specialists with hospitalized children and child development and family systems theories adapted from “Resiliency model” of family stress, adjustment and adaptation | Improve parenting quality and self-efficacy and increase family communication about illness | Psycho-educative, reflective and skill-building elements | Key contents - Parent Session 1: Intervention started with family consultation session - Children Sessions: Six weekly (1hr/session) - Session 1: Getting to know interventionist and initial assessment - Session 2: Education about cancer - Session 3: Help child express felling - Session 4 and 5: Educate child on stress and coping technics - Session 6: Guide child to develop hope for the future - Session 7: Tour to cancer treatment center - # After each session letters sent to parents - Parent Session 2: Parent consultation | Nine session (1hr/ session) | Face-to-face in the family | Certified child life specialists (CCLS) |
| Preventive Counselling Project (Thastum et al., 2006) | Chong et al., 2024; Strandh et al., 2023; Zhao et al., 2024 | Not mentioned | Family-focused | Ill parents + Children + Healthy parents | Not mentioned | Enhance parenting competence, support the parents in age-appropriate communication and support the parents’ use of possible network | Psycho-educative, reflective and counselling elements | Key contents 1.First session (To parents) -Details: Set goals and frameworks of the sessions afterwards -Goal: Match the expectations of counselling between parents and counsellors 2. Second to Fifth session (To parents and children): -Details: Family counselling under the frameworks discussed in first session -Goal: Facilitate open communication in the family 3. Sixth session (To children): -Details: Offer group counselling with other children -Goal: Enhance children’s knowledge on parental cancer and active coping style | 5–6 sessions; period not mentioned | Face to face with the family, at community setting | 2 trained counsellors + 4 trained psychotherapists (qualification of counsellors and psychotherapists not mentioned) |
| ‘Family Talks in Cancer Care’ Program (Bugge et al. 2008, 2009) | Ellis et al., 2017; Inhestern et al., 2016; Strandh et al., 2023; Zhao et al., 2024 | Palliative stage | Family-focused | Parents  Children  Whole family | Libo and Griffith’s (1996) coping theory for children and the Allison et al.’s (2003) Family Resilience Theory | Prevent psychosocial problems; Promote coping; Improve communication; Improve knowledge about cancer; Help the family plan for the future | Psycho-educative and skill-building elements | Not mentioned | 5 sessions over  6 weeks | Face-to-face with the family; Fully manualized child-centred  family support groups | Nurses, sociologist and art therapist |
| Parent Guidance Intervention (Christ et al., 2005) | Ellis et al., 2017; Inhestern et al., 2016 | Terminal cancer | Parent-focused  (healthy) | Well parent  Children  Whole family | Not mentioned | Prepare the family for death, and  Improve their communication and  interaction skills  Facilitate children’s adjustment  to disease /death, support the  well parent to deal with own  grief, support continuance of  well parent's parental functioning | Not mentioned | Not mentioned | 6 sessions pre- and post-death,  60–90 min, over  12 months | Therapeutic interviews in the  home environment | Not mentioned |
| The Bear Essentials’ (Greening, 1992) | Ellis et al., 2017; Inhestern et al., 2016 | Not mentioned | Child-focused | Parents  Children | Not mentioned | Increase understanding of children’s  perceptions of illness, separation,  and loss; Providing a supportive environment  for parents and children to discuss  concerns and develop coping  strategies  Support families in  understanding each other and  coping; provide supportive  environment to discuss  concerns | Reading,  craft and play; discussion group | Children participate in  child-centred therapeutic  activities.  Parents participate  in a concurrent semi-structured  discussion group | Monthly,  90 min sessions | Support groups | Not mentioned |
| ‘Quest’ (Heiney & Lesesne, 1996) | Ellis et al., 2017; Inhestern et al., 2016 | Not mentioned | Child-focused | Parents  Children | Not mentioned | To facilitate positive coping; Improve family communication; Improve knowledge about cancer; Normalize emotions/experiences; Reduce isolation | Activities include, dinner, getting  to know each other game,  educational discussion and  hospital tour | Not mentioned | Biannual evening  program, 2 hours | Parents participate in pre/post –  program interviews. Children  are divided into groups by age | Not mentioned |
| Getting well together (John et al., 2013) | Ellis et al., 2017; Geertz et al., 2023; Inhestern et al., 2016; Strandh et al., 2023 | Stage cancer 0-III | Family-focused | Parents (mothers with breast cancer)  Children  Parent–child  dyad | Not mentioned | Support the family system,  prevent at risk children from  developing serious emotional  and behavioural problems | Psycho-educative, resource oriented discussion and counselling elements | Resource-oriented positive psychology, stress and coping research, systemic solution focused therapy, and the COSIP (Children of Somatically Ill Parents) manual | 11 sessions over 3 weeks | Inpatient rehabilitation program  incorporating fully manualized  child-centred group intervention,  child care and oncological  rehabilitation for the mother | Not mentioned |
| The Children’s Summer Programme (Naudi, 2002) | Ellis et al., 2017 | Advanced cancer | Child-focused | 25 children | Not mentioned | Provide respite to parents and to Provide children quality time in a safe environment Provide opportunities to interact with other children | Activities include picnics, boat trips, visits to theme parks, cinema or other places of interest | Not mentioned | 3 days/week for 8 weeks from 8 am to 2 pm in summer holidays | Summer camp | Not mentioned |
| ‘Kids Can Cope’ (Taylor-Brown et al., 1993) | Ellis et al., 2017; Inhestern et al., 2016 | Not mentioned | Child-focused | Parents  Children | Not mentioned | Increase knowledge about cancer; Provide a supportive environment for children to express their feelings  and experiences; Normalize emotions/experience; Enhance adaptive coping skills; Guide future research and program  development | Education based and therapeutic  activities | Not mentioned | 6 weekly sessions  + information session for  parents | The children are divided into  groups of 4–8 members | Not mentioned |
| Family Matters (Werner-Lin & Biank, 2009) | Ellis et al., 2017 | Not mentioned | Family-focused | Parents and their children (5–11 years) | Not mentioned | Normalize emotions/experiences; Reshape the family system to maximize resources and build on areas of vulnerability | Psycho-educational programs, support groups, family therapy sessions, and social events | Not mentioned | Variable | Not mentioned | Not mentioned |
| Psychoeducational Group Interventions for Families with Parental Cancer (Heinemann & Simeit, 2017) | Geertz et al., 2023 | Not mentioned | Family-focused | Families with  children aged 5–14  and a parent with  cancer | Not mentioned | Not mentioned | Not mentioned | Not mentioned | 12 weekly sessions (+3  additional if  necessary) each  90 min | Group Sessions | Not mentioned |
| Psychosocial Well-Being of Young People Who Participated in a Support Group Following the Loss of a Parent to Cancer (Olsson et al., 2017) | Geertz et al., 2023 | Not mentioned | Grieving after the loss of a parent to cancer | Adolescent and Young Adults who had lost a  parent to cancer  the months before. | Stroebe and Schuts dual  process model and Hult  and Waads salutogenic,  health promoting  methodology | Not mentioned | Not mentioned | Not mentioned | Weekly 2 h sessions;  Duration: 10 weeks | Not mentioned | Professional group  leaders |
| Good Grief program for young people bereaved by familial cancer (Patterson et al., 2021) | Geertz et al., 2023 | Not mentioned | Grieving after the loss of a parent to cancer | Adolescent and Young Adults bereaved by  the death of a  parent or sibling  from cancer | Stroebe and Schuts dual  process model, self-  compassion, continuing  bonds, constructivism  and meaning making  approaches to grief were  used in the development  of the program | Camp-based  program focused  on meeting coping,  social support, and  respite needs of  AYAs bereaved by  familial cancer. | Not mentioned | Not mentioned | One time 3-day-camp  with 6 psychosocial  sessions and  recreational  activities (e.g. water  sports, group games) | Camp-site | Not mentioned |
| Family Focused Grief Therapy (Kissane et al., 2006, 2007) | Inhestern et al., 2016 | Terminal cancer | Family-focused | Whole family | Not mentioned | Optimize cohesion,  communication, and handling of  conflict, promote the sharing of  grief and mutual support | Not mentioned | Not mentioned | 4–8  sessions before and after  death, 90 minutes | Not mentioned | Not mentioned |
| Short-term psychoeducational intervention (Hoke, 1997) | Inhestern et al., 2016 | Not mentioned | Family-focused | Whole  family | Not mentioned | Share concerns and talk about  disease; increase understanding  and support within families | Not mentioned | Not mentioned | About 6  sessions | Not mentioned | Not mentioned |
| Art-therapy program for parents (Weiß et al. 2005) | Inhestern et al., 2016 | Not mentioned | Parent-focused | Younger patients  with and without  children – group intervention | Not mentioned | Creating something, reassure  self-confidence, design a book  to support communication with  children | Not mentioned | Not mentioned | 22  weekly sessions - 90  minutes | Group setting | Not mentioned |
| Being a parent and coping with cancer (Hasson-Ohayon & Braun, 2011) | Inhestern et al., 2016 | Stage cancer 0-III | Parent-focused | Patients with  children undergoing  chemo - | Not mentioned | Empower the patient and  spouse in their parenting, help  the parents to help their children  to adjust and cope | Not mentioned | Not mentioned | 4 sessions/ 1 day  workshop | Group setting | Not mentioned |
| For kids only (Bedway & Smith, 1997) | Inhestern et al., 2016 | Not mentioned | Child-focused | Children  (preschool-  adolescents) | Not mentioned | Education, support & screening  of children; provide safe  environment | Not mentioned | Not mentioned | 1-day workshop | Group setting | Not mentioned |
| School-based support group (Call, 1990) | Inhestern et al., 2016 | Not mentioned | Child-centred group | School-aged  children 6–12 years | Not mentioned | Develop coping skills, create  safe environment, share  feelings, keep on with activities,  educate about disease | Not mentioned | Not mentioned | 10 weekly sessions, 50–  55 minutes | Group setting | Not mentioned |
| FAMOCA (Family online counselling for families with parental cancer) (Denzinger et al., 2019) | Malinowski et al., 2025; Strandh et al., 2023; Zhao et al., 2024 | Early stage | Family-focused | Children and  parents | Cognitive-behavioural theory | Improvement of child and parental adjustment and family functioning | Psycho-educative elements | 4 modules based on  CBT techniques to  foster adaption  and coping strategies.  Uses the “minimal contact” concept (autonomous use of the  program combined  with regular  feedback by a professional). Most of the modules  were designed  for parents to  work with their  younger children  (3–11), though some designed for  children to complete  on their own | 4 modules (4 weeks each) | Web‐based intervention  Program, Online individually | Psychologist |
| Skills Training on the Quality of Life Among the Daughters of Mothers With Breast Cancer (Khanjari et al., 2020) | Malinowski et al., 2025 | Active  treatment, receiving  chemotherapy | Family-focused | Adolescent daughters of ill mothers | Not mentioned | Improve the  coping skills of  daughters of  mothers with  breast cancer | The  training included  a workshop,  simulation and  follow‐up program  designed to help | Not mentioned | Three 1 ¾ hour  sessions and the  4th session was 2 h | In‐person group conducted in a hospital setting | Not mentioned |
| Healing through Arts” (HeARTS) (Ong et al., 2023) | Malinowski et al., 2025 | Grieving after the loss of a parent to cancer | Child-centred | Bereaved children whose parent had died from cancer within the previous 2 years | Not mentioned | Facilitate the  expression of feelings  associated  with grief and to  memorialize the  deceased parent | Art activities to help facilitate the expression of feelings associated with grief and to memorialize the deceased parent | Not mentioned | 3‐day | In‐person  group | Art therapist |
| Unnamed intervention (Davis Kirsch et al., 2003) | Strandh et al., 2023 | Active  treatment, receiving  chemotherapy | Family-focused | Mother and child | A developmental–contextual  model of parenting, coping  theory, and social cognitive  theory | Enhance the  interaction between  mother and child  during the cancer  treatment | Not mentioned | Psycho-educative elements  and homework assignments | Not mentioned | Face-to-face with the family | Not mentioned |
| Family-SCOUT (Dohmen et al., 2021) | Strandh et al., 2023 | Not mentioned | Family-focused | Families with minors suffering from parental cancer | The COSIP (Children of  Somatically Ill Parents) manual | Provide support  for families with  minors suffering  from parental cancer | Counselling session, support  guidance, coordination of care  and discussion elements over  the entire course of disease | Not mentioned | Not mentioned | Face-to-face and via  telephone with the family | Social workers and nurses |
| A short-time counselling intervention (Erhbar et al., 2022) | Strandh et al., 2023; Zhao et al., 2024 | Not mentioned | Family-focused | Children and parents affected by parental cancer | The COSIP (Children of Somatically Ill Parents) manual | Enhance adjustment in children and parents affected by parental cancer by fostering open communication, family cohesion, affective involvement, and adaptive coping | Not mentioned | Evaluation of the family situation, psycho-education, emotion regulation and assignments in six sessions over six weeks | Not mentioned | Not mentioned | Not mentioned |
| A brief, cost-effective family-focused intervention (Fife et al., 2017) | Strandh et al., 2023 | Active  treatment | Family-  focused intervention | Families with  a parent with  cancer | None | Reduce emotional  distress, facilitate  supportive  functioning within  the family and  promote adaptive  coping | Not mentioned | Psychoeducation and  counselling. | In two sessions for  the patient and caregiver and  one session for the caregiver  over the time period from  before hospitalization through  4 months post discharge | Face-to-face and via  telephone individually | Not mentioned |
| Being a Parent and Coping with Cancer (PCWC) (Hasson-Ohayon & Braun, 2011) | Strandh et al., 2023; Zhao et al., 2024 | Not mentioned | Parent-focused | Patients and spouses | None | Empower the patients and spouses in their parenting role, and to help the parents help their children to adjust and cope | Not mentioned | Psycho-educative and discussion elements in 4 modules. | Not mentioned | Face-to-face in groups | Not mentioned |
| Information booklet (Melchiors et al., 2022) | Strandh et al., 2023; Zhao et al., 2024 | Not mentioned | Parent-focused | Parents | None | Address a lack of information on age-specific communication and developmental aspects of children and an overview of local support offers for affected families | Not mentioned | Psycho-education | Not mentioned | Text-based: booklet | Not mentioned |
| Families Addressing Cancer Together (FACT) (Park et al., 2022) | Strandh et al., 2023; Zhao et al., 2024 | Not mentioned | Parent-focused | Parents | The Health Disclosure Decision-Making Model and Social Cognitive Theory | To address the communication needs of parents with cancer | Not mentioned | Psycho-education and skill-building elements in 5 modules. | Not mentioned | Online individually | Not mentioned |
| Enhancing Parenting in Cancer (EPIC) (Stafford et al., 2017, 2021) | Strandh et al., 2023; Zhao et al., 2024 | Not mentioned | Parent-focused | Parents | Attachment and social cognitive theory | Improve parenting efficacy and promote family communication, thereby decreasing parental stress and psychological morbidity | Not mentioned | Psycho-educative and reflective elements | Not mentioned | Not mentioned | Not mentioned |
| A brochure (Turner et al., 2007) | Strandh et al., 2023 | Advanced cancer | Parent-focused | Mothers with  advanced breast  cancer | Not mentioned | Encourage parents  to feel more  confident talking  about cancer with  their children, give  suggestions about  coping strategies,  and to give guidance  about available  resources | Not mentioned | Information on how to talk  to children in the format och  questions and answers | Not mentioned | Text-based: brochure | Not mentioned |
| The Fuelbox “Young Next of Kin” (FYNK) (Hauken & Farbrot, 2022) | Zhao et al., 2024 | Not mentioned | Not mentioned | Adolescents, parents and professionals | Parent-focused | Not mentioned | Not mentioned | Not mentioned | Not mentioned | Not mentioned | Not mentioned |
| Intervention group: an information booklet (Lienard et al., 2022) | Zhao et al., 2024 | Not mentioned | Parent-focused | 60 parents, 31 were  intervention group | Not mentioned | Not mentioned | Not mentioned | Not mentioned | 4-session intervention | An  information booklet | Not mentioned |
